# Distribution and Removal of Pharmaceuticals in Liquid and Solid Phases in the Unit Processes of Sewage Treatment Plants

**DOI:** 10.3390/ijerph17030687

**Published:** 2020-01-21

**Authors:** Junwon Park, Changsoo Kim, Youngmin Hong, Wonseok Lee, Hyenmi Chung, Dong-Hwan Jeong, Hyunook Kim

**Affiliations:** 1 Department of Environmental Infrastructure Research, National Institute of Environmental Research, Ministry of Environment, 42 Hwangyeong-ro, Seo-gu, Incheon 22689, Korea; newjjun@korea.kr (J.P.); scvcontrol@korea.kr (C.K.); boystone@korea.kr (W.L.); hyenmic@korea.kr (H.C.); 2 Technical Research Center, Shimadzu Scientific Korea, 145 Gasan digital 1-ro, Geumcheon-gu, Seoul 08056, Korea; ymhong@shimadzu.co.kr; 3 Department of Environmental Engineering, University of Seoul, 163 Seoulsiripdaero, Dongdaemun-gu, Seoul 02054, Korea

**Keywords:** pharmaceuticals, biological treatment processes, mass balance, sewage treatment plant

## Abstract

In this study, we analyzed 27 pharmaceuticals in liquid and solid phase samples collected from the unit processes of four different sewage treatment plants (STPs) to evaluate their distribution and behavior of the pharmaceuticals. The examination of the relative distributions of various categories of pharmaceuticals in the influent showed that non-steroidal anti-inflammatory drugs (NSAIDs) were the most dominant. While the relative distribution of antibiotics in the influent was not high (i.e., 3%–5%), it increased to 14%–30% in the effluent. In the four STPs, the mass load of the target pharmaceuticals was reduced by 88%–95% mainly in the biological treatment process, whereas the ratio of pharmaceuticals in waste sludge to those in the influent (*w*/*w*) was only 2%. In all the STPs, the removal efficiencies for the stimulant caffeine, NSAIDs (acetaminophen, naproxen, and acetylsalicylic acid), and the antibiotic cefradine were high; they were removed mainly by biological processes. Certain compounds, such as the NSAID ketoprofen, contrast agent iopromide, lipid regulator gemfibrozil, and antibiotic sulfamethoxazole, showed varying removal efficiencies depending on the contribution of biodegradation and sludge sorption. In addition, a quantitative meta-analysis was performed to compare the pharmaceutical removal efficiencies of the biological treatment processes in the four STPs, which were a membrane bioreactor (MBR) process, sequencing batch reactor (SBR) process, anaerobic–anoxic–oxic (A2O) process, and moving-bed biofilm reactor (MBBR) process. Among the biological processes, the removal efficiency was in the order of MBR > SBR > A2O > MBBR. Among the tertiary treatment processes investigated, powdered activated carbon showed the highest removal efficiency of 18%–63% for gemfibrozil, ibuprofen, ketoprofen, atenolol, cimetidine, and trimethoprim.

## 1. Introduction

With rising living standards, the introduction of new chemical substances, and the development of medical technology, new trace contaminants are being detected in water environments, which has drawn significant social attention [1,2]. Among various types of trace contaminants, pharmaceuticals such as antibiotics, analgesics, and non-steroidal anti-inflammatory drugs (NSAIDs) are mainly used to treat human and animal diseases, develop the growth accelerator industry, and improve immunity. Long-term exposure to these substances, even in trace amounts in the environment, can negatively affect aquatic ecosystems and result in chronic toxicity, endocrine disruption, and antibiotic resistance. Reports have also indicated potential danger to the human body [3,4]. While pharmaceuticals consumed by humans and animals are partially metabolized, metabolites are secreted in the form of urine or feces and enter sewage treatment plants (STPs) [5]. However, most STPs using conventional activated sludge (CAS) treatment processes are not designed to remove trace contaminants such as pharmaceuticals. Therefore, these substances are discharged without being completely removed; this is known to be the main cause of the detection of pharmaceuticals in water environments [6,7,8]. Pharmaceuticals can be partially removed through advanced treatment processes such as ozone oxidation and powdered activated carbon (PAC) treatment, which are designed for disinfection and water reuse. Certain pharmaceuticals can be removed with high efficiency in biological treatment processes, the main removal mechanisms being biodegradation and sludge sorption [9,10,11]. The pharmaceuticals removed from STPs are characterized by factors related to the properties of the target compounds, such as hydrophilicity, hydrophobicity, and biodegradability, as well as factors related to the processes performed in the STP, such as hydraulic retention time, sludge retention time (SRT), redox conditions, temperature, and pH [12].

Nevertheless, previous studies calculated the removal efficiency of pharmaceuticals by STPs simply based on the concentrations of the chemicals in influent sewage and effluent. However, recent studies quantitatively analyzed not only the influent of STPs, but also the substances adsorbed to the solid phase, such as suspended solids (SS) composed of organic solids, clays, and silts in process water and waste sludge and activated sludge in the bioreactors [13,14]. Analytical methods for quantifying a variety of pharmaceuticals at trace levels in complex matrices were also developed and applied in our previous works [15,16]. This enables a more accurate assessment of the behavior and effective removal of pharmaceuticals. In particular, studies have evaluated the removal characteristics of pharmaceuticals by analyzing the mass balance of unit processes in STPs using various treatment processes, such as CAS, anaerobic–anoxic–oxic (A2O), membrane bioreactor (MBR), aerated lagoon, and oxidation ditch processes [14,17,18,19,20]. However, the results of previous studies were not comprehensive. For a more accurate evaluation of the fate of pharmaceuticals in a sewage system, a more detailed monitoring study should be designed and carried out, simply because a variety of unit processes are installed, complicatedly connected, and operated in a full scale STP. In addition, each STP is designed differently; namely, unit treatment processes differ across various STPs. According to statistical data, there are 604 STPs with a treatment capacity of 500 m^3^/d or more in South Korea. Based on the type of biological treatment process, these plants can be classified into plants using sequencing batch reactors (SBR) (35%), A2O (26%), MBBR (23%), and MBR (9%) [21]. Therefore, there is a need for comprehensive information on the degree of removal of pharmaceuticals by each treatment process. In addition to the main treatment processes consisting of primary treatment, secondary treatment, and tertiary treatment, evaluating the distribution characteristics of pharmaceuticals (in reject water, internal and external return sludge, waste sludge, etc.) can improve the overall understanding of the behavior of these substances in STPs.

Accordingly, this study investigated the contents of the target substances, namely 27 types of pharmaceuticals including antibiotics, NSAIDs, and antiarrhythmic agents, in the liquid and solid phase samples obtained from each unit operation of STPs. We then calculated the mass load of each substance, classified them according to their pharmacological use, and analyzed their fate for each unit operation. The target STPs were selected from plants in which the four types of treatment processes mentioned above comprised 93% of all the sewage treatment processes. We investigated the main removal mechanisms of the target substances through the analysis of their mass balance, and accordingly compared the removal efficiencies of the sewage treatment processes through meta-analysis. In addition, we evaluated the removal efficiencies of the tertiary treatment processes, including full-scale coagulation, ultraviolet (UV) treatment, and PAC treatment, in removing pharmaceuticals, and evaluated their degree of contribution to the overall treatment efficiency.

## 2. Materials and Methods

### 2.1. Target Compounds

Based on the marketing data of pharmaceuticals, 57 types of pharmaceuticals were initially selected as target compounds in our preliminary study. Out of the 57 compounds, we selected 27 compounds as the targets in this study based on their influent concentrations and detection frequencies. In total 27 types of pharmaceuticals, including 11 antibiotics, 6 anti-inflammatory/NSAIDs, 2 antiarrhythmic drugs, and 1 stimulant were selected as the target substances. Table 1 shows the physicochemical properties of the substances. For the standard substances and reagents used in the analysis, 10 mg/L of a standard solution of 98% or higher purity purchased from Sigma-Aldrich (Saint Louis, MO, USA) and Fluka (Seelze, Germany) was prepared and stored in a freezer at −20 °C or below. It was diluted and used on the day of the analysis. Twenty types of isotopically labeled standards (acetaminophen-d4, salicylic acid-d4, atenolol-d7, caffeine-13C3, carbamazepine-d10, cimetidine-d3, ciprofloxacin-d8, diclofenac-13C6, diphenhydramine-d3, erythromycin-d6, gemfibrozil-d6, ibuprofen-d3, iopromide-d3, ketoprofen-d3, naproxen-d3, ofloxacin-d3, propranolol-d7, sulfadimethoxine-13C6, testosterone-d3, and trimethoprim-d9) were purchased from Sigma-Aldrich and Toronto Research Chemicals (Toronto, ON, Canada).

### 2.2. Specifications of Sewage Treatment Plants

We selected four STPs in Gyeonggi-province, South Korea, using MBR, SBR, A2O, or MBBR as a main sewage treatment process. Samples were collected three times, i.e., in June–July 2018, September–October 2018, and December 2018–January 2019, during normal operation of each STP. The primary sludge, waste sludge, and reject water were collected using the grab sampling method, whereas the 24 h composite sampling method was used for all other samples. In the STPs under study, the wastewater generating from the sludge-thickening and -dewatering processes is re-entered into their main-stream processes. The returned wastewater is referred to as reject water and contains a larger amount of pollutants such as nitrogen and phosphorus. Pharmaceutical compounds are also present at higher concentrations in the reject water, possibly having a negative impact on the treatment stability of a STP. Therefore, the pharmaceutical concentrations in the thickening water and dewatering water in STP A, washing water and dewatering water in STP B, and dewatering water in STP C were investigated.

Equal volume discrete sample aliquots (250 mL) were collected manually from predetermined points of each STP (Figure S1), using amber glass bottles previously rinsed with acetone and dried at 100 °C (i.e., 6 samples over the course of a day, at 4-h intervals). Before each sample was collected, the respective amber bottle was thoroughly rinsed with wastewater. Each collected aliquot was preserved in an icebox until the sixth sample was collected. Once all the aliquots were collected, they were put into a 2 L glass bottle, which was then put into the icebox for the transport to the laboratory. All samples were stored at 4 °C, and the samples were analyzed within 48 h. Table S1 shows the influent characteristics and operating conditions. Table S2 shows the treatment systems of the target STPs. STP A used the MBR process using a polyvinylidene fluoride (PVDF) membrane with 0.04 μm pores; the MBR effluent was discharged directly to the ocean without tertiary treatment. STP B used the SBR process; its tertiary treatment used coagulation and disk filtration for treatment of total phosphorus and UV light for disinfection. STP C used a four-stage biological nutrient removal (BNR) system, and its tertiary treatment used coagulation and sedimentation. STP D used a treatment technique that placed the fluidized bed in an aerobic tank and employed a post-denitrification process. In the tertiary treatment, the effluent of the bioreactor was treated for total phosphorus using high-speed coagulation/sedimentation, and it was supplied as urban reuse water after PAC treatment. Only domestic sewage produced from residential area is treated in the target STPs. It contains human pharmaceuticals which are excreted in the urine as well as various pollutants such as organic matter, nutrients, and pathogens. All STPs do not receive any industrial and agricultural wastewater. They have advantages of removing a variety of pollutants and operated stably whilst meeting effluent quality standards.

### 2.3. Analytical Methods

As pharmaceuticals may exist in a liquid phase or solid phase in STPs, different pretreatment processes were applied to the two types of samples. A schematic diagram of analytical methods for determination of pharmaceuticals is shown in Figure S2. First, a liquid sample was filtered using a 0.45 μm pore size glass-fiber filter, and 900 μL of a filtered sample was collected in a 2 mL LC-MS/MS vial. Then, 1% formic acid (100 μL; Wako Pure Chemical, Osaka, Japan), ethylenediaminetetraacetic acid disodium salt dehydrate (Na_2_EDTA; 10 μL; Sigma-Aldrich, Saint Louis, MO, USA) at a concentration of 40 mg/mL, and isotopically labeled standards (10 μL) at a concentration of 10 μg/L were added. The quick, easy, cheap, effective, rugged, and safe (QuEChERS) method was modified to extract target compounds from the solid phase samples, the details of which are shown in Text S1. The extraction step of the QuEChERS method is based on partitioning through salting-out extraction; acetonitrile is used as the extraction solvent, and the interfering substances contained in the extract are removed in the cleanup step after extraction. The quantities of SS in the influent, primary effluent, secondary effluent, and effluent were less than 0.5 g; if filtered, it looked muddy. Therefore, a 0.45 μm glass-fiber filter (47 mm; Hawach Scientific, Xi′an, China) was used to extract pharmaceuticals from the SS in a sample. The solid-absorbed filter was placed in a 50 mL glass centrifuge tube, and 1% acetic acid (5 mL; Sigma-Aldrich, Saint Louis, MO, USA), acetonitrile (10 mL; J.T.Baker, Phillipsburg, NJ, USA), anhydrous sodium sulfate (2 g; Wako Pure Chemical, Osaka, Japan), Na_2_EDTA (0.2 mL) at a concentration of 40 mg/mL, and isotopically labeled standards (100 μL) at a concentration of 100 μg/L were added and mixed using a vortex for 1 min. Then, the mixture was subjected to solid-liquid separation at 4000 rpm for 5 min. One milliliter of the acetonitrile layer was collected in a glass tube, concentrated using a nitrogen concentrator, and dissolved in a solvent mixture of methanol and 0.1% formic acid at a ratio of 1:9. Finally, the sample was filtered using a syringe filter with a 0.45 μm pore size PVDF membrane (Advantec, Tokyo, Japan) to create the final sample.

The sludge in the solid phase was subjected to solid-liquid separation at 3000 rpm for 5 min using a centrifuge. The supernatant obtained after centrifugation of the sludge was filtered using a syringe filter with a 0.45 μm pore size PVDF membrane and processed using the same procedure as that for the liquid phase sample. One gram of wet sludge remaining in the glass centrifuge tube was collected, and the solid samples were extracted by the same process as that for the SS. The analysis was performed in triplicate; Table S3 shows the details of the parameters used. The peaks were identified and quantified using high-performance liquid chromatography (HPLC; Nexera X2, Shimadzu, Japan) and mass spectrometry (LCMS-8050, Shimadzu, Japan). ACE 5 C18-PFP (150 mm × 2.1 mm) and MAYI-ODS (G) (2.0 mm × 10 mm) were used for the HPLC and online solid phase extraction columns. The details of online SPE-LC/MS/MS analysis are shown in Text S2. The MS analysis was performed by using negative electrospray ionization (ESI) mode for four types of substances (acetylsalicylic acid, diclofenac, ibuprofen, and gemfibrozil), whereas other substances were ionized in the positive ESI mode.

### 2.4. Quality Control

The limit of detection and limit of quantification (LOQ) were calculated to have a S/N ratio of 3 or more and 10 or more, respectively, on the chromatogram [22]. Table S4 shows the quality control results for the LOQ and recovery rate for the liquid and solid phases of each substance. The LOQ values of the liquid and solid phase were 1–31.6 ng/L and 0.6–4 ng/g, respectively. The target substance recovery was 91%–117% for the liquid phase samples and 61%–137% for the solid phase samples. The intra-day repeatability was estimated by analyzing seven replicate samples at three different levels and it showed a relative standard deviation of less than 10% on average. The intra-day reproducibility was determined by the analysis of seven replicates on three consecutive days; it was within 20% for each of the target compounds. The recoveries of some compounds at a certain level were less than 70% (e.g., ciprofloxacin, gemfibrozil, and sulfadimethoxine) or over 130% (e.g., clarithromycin, ibuprofen, and iopromide). Recoveries outside the acceptable range might be associated with the matrix complexity of a samples and/or physicochemical properties of target compounds, such as acidity, solubility, and polarity. Surrogate recovery, which was used to estimate loss in the extraction stage during pretreatment, was 60%–130%, which was similar to the recovery rate suggested in previous studies [19,20]. In general, the results of the method validation indicate that the method used in this study is adequate for the simultaneous analysis of multiple pharmaceuticals in both the liquid phase and solid phase.

### 2.5. Calculations of Mass Balance and Standardized Removal Efficiency

We calculated the daily mass load in the unit operation of each STP and used Equation (1) to analyze the mass balance [9].

M_influent_ = M_effluent_ + M_biodegradation_ + M_sorption_, (1)

where M_influent_ (g/d) = flow rate (m^3^/d) × influent concentration of each pharmaceutical (ng/L) × 10^−6^; M_effluent_ (g/d) = flow rate (m^3^/d) × effluent concentration of each pharmaceutical (ng/L) × 10^−6^; M_sorption_ (g/d) = sludge production rate (m^3^/d) × sludge concentration of each pharmaceutical (ng/L) × 10^−6^; and M_biodegradation_ (g/d) was calculated from the mass loads of the influent, effluent, and sorption.

In the biological treatment process of the STP, it may be difficult to accurately evaluate the removal of pharmaceuticals owing to various factors such as the characteristics of the substance and the operating conditions of the treatment process. Meta-analysis has proven very useful for integrating and interpreting large amounts of data, and has been extensively applied in various fields such as medicine, ecology, and toxicology [23]. This study used the meta-analysis method proposed in previous literature to quantitatively evaluate the uncertainty in the removal efficiency of different compounds by each treatment process. Equation (2) was used to calculate the standardized removal efficiency (SRE) [23,24].

(2) $$SRE=\frac{\left(\left(x-\mu \right)/\sigma \right)\times n}{N},$$

where *x* is the removal efficiency of each substance, *μ* is the mean removal efficiency of the substance in all sewage treatment processes, *σ* is the standard deviation of the substance in all sewage treatment processes, *n* is the number of data points in each sewage treatment process, and *N* is the total number of data points in all sewage treatment processes.

### 2.6. Statistical Analysis

All statistical analyses were performed using GraphPad Prism 5.0 (GraphPad Software, Inc., San Diego, CA, USA). For determination of significant variance between treatment processes and removal efficiencies of pharmaceuticals, a one-way analysis of variance (ANOVA) with Tukey’s multiple-comparison post hoc test was performed. Significant variance was achieved with *p* < 0.05.

## 3. Results and Discussion

### 3.1. Occurrence of Pharmaceuticals in Different Unit Processes of Sewage Treatment Plants

#### 3.1.1. Concentrations of Pharmaceuticals in Sewage

**Figure 1:**
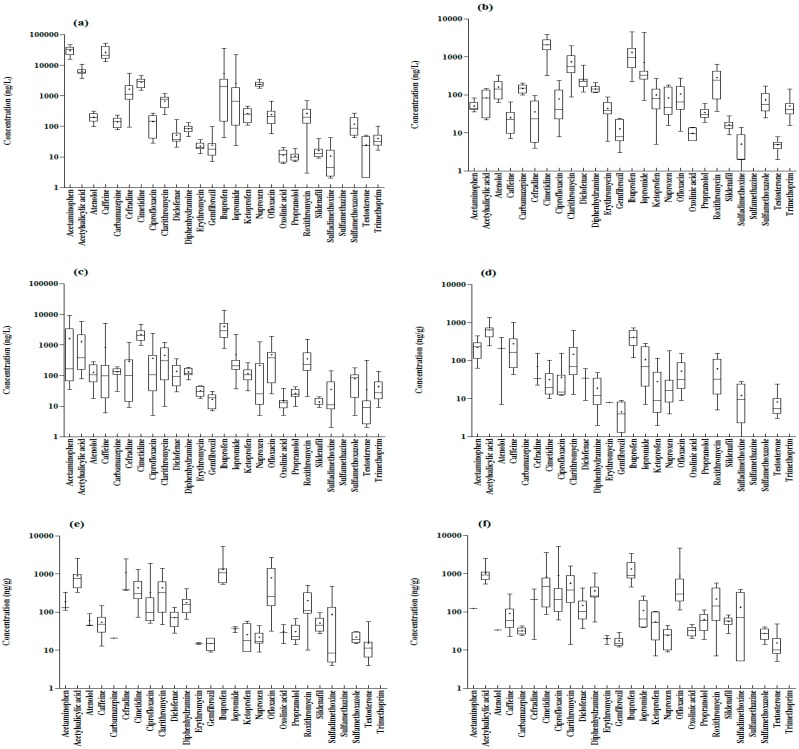
Concentrations of 27 Pharmaceuticals in (**a**) influent, (**b**) effluent, (**c**) reject water, (**d**) suspended solids, (**e**) activated sludge, and (**f**) waste sludge of 4 STPs.

Among the 27 types of substances in the influent, 16 showed 100% detection frequency and 25 showed at least 50% detection frequency. Acetaminophen (NSAID) had the highest concentration at 30,000 ng/L, followed by caffeine (26,000 ng/L), acetylsalicylic acid (6500 ng/L), ibuprofen (5300 ng/L), and cimetidine (2800 ng/L) (Figure 1a). Previous studies also showed high concentrations of these substances, and non-prescription NSAIDs, namely acetaminophen and ibuprofen, have been reported at higher levels than other NSAIDs [25,26,27]. In the case of antibiotics, the mean concentrations of cefradine, clarithromycin, roxithromycin, ofloxacin, and ciprofloxacin were 1600 ng/L, 670 ng/L, 270 ng/L, 240 ng/L, and 140 ng/L, respectively. Relatively high concentrations of macrolide and fluoroquinolone were detected. The high detection rates of these substances in the influent were related to their consumption; according to statistics from 2000 to 2015, fluoroquinolone and macrolide had the third and fourth highest consumption rates for antibiotics worldwide [28]. In the South Korean antibiotic market, macrolide (12%) and fluoroquinolone (10%) were ranked the third and fourth after penicillin (38%) and cephem (26%) [29]. Similar to the results of this study, high levels of fluoroquinolone and macrolide antibiotics were detected in wastewater treatment plant (WWTP) influent in Europe, North America, and Asia [27]. Sulfamethoxazole showed the highest concentration among sulfonamide antibiotics at 120 ng/L, but the concentration of sulfamethazine was below the LOQ. Certain substances such as testosterone, erythromycin, sildenafil, oxolinic acid, sulfadimethoxine, and propranolol were present in the mean concentration range of 10–30 ng/L, which was low when compared with the concentrations of other substances.

The detection frequency of eight types of substances (atenolol, carbamazepine, cimetidine, diclofenac, naproxen, ofloxacin, sulfamethoxazole, and trimethoprim) was 100% in the effluent, and the detection frequency of 25 types of substances, excluding oxolinic acid and sulfamethazine, was 50% or greater. Sulfamethazine was found to be below the LOQ, which was similar to that in the influent. Cimetidine showed the highest concentration of 2100 ng/L; the substances in decreasing order of their concentrations were ibuprofen (1300 ng/L), clarithromycin (740 ng/L), iopromide (730 ng/L), roxithromycin (290 ng/L), and diclofenac (250 ng/L) (Figure 1b). A mean concentration range of 100–200 ng/L was found for atenolol, carbamazepine, diphenhydramine, ofloxacin, and ketoprofen, whereas concentrations of 16 ng/L, 13 ng/L, 10 ng/L, 5.1 ng/L, and 5.0 ng/L were found for sildenafil, gemfibrozil, oxolinic acid, sulfadimethoxine, and testosterone, respectively. Although the concentrations of these substances in the effluent were low, they were higher than the LOQ.

#### 3.1.2. Concentrations of Pharmaceuticals in Reject Water

With the exception of sulfamethazine, 26 types of substances were detected at a frequency of at least 50%, and 12 were detected with 100% frequency. Ibuprofen, cimetidine, acetaminophen, and acetylsalicylic acid were detected at concentrations of 1000 ng/L or more, and 13 types of substances including caffeine, ofloxacin, and iopromide showed a concentration range of 100–1000 ng/L (Figure 1c).

We evaluated the ratio of the reject water concentration to the influent concentration (R/I ratio) (Figure S3). If this ratio is high, it means that the amount of a certain compound returned from the treatment process is higher than that entering into the STP. Testosterone, oxolinic acid, sulfadimethoxine, and propranolol showed high R/I ratios despite low reject water concentrations, as these substances were not detected at high concentrations in the influent. The R/I ratios of ciprofloxacin, roxithromycin, and ofloxacin were 3.1, 3.0, and 2.6, respectively, thereby indicating that the reject water concentration was higher than that of the influent. This suggested a high rate of return of these antibiotics to the STP through the reject water. In contrast, caffeine, acetaminophen, naproxen, acetylsalicylic acid, and cefradine were detected at high levels in the influent, but their R/I ratios were low. Ibuprofen showed the highest R/I ratio of 24.3, thereby indicating that the concentration of ibuprofen measured in the two influent samples was lower than the mean by two orders of magnitude, which increased the ratio (the R/I ratio excluding the two samples was 2.2). Many substances had large R/I ratio fluctuations. This concentration fluctuation might be attributed to the intermittently-occurring nature of the reject water in the STPs. More importantly, the quality of the reject water is directly influenced by the anaerobic digestors (ADs) operated by the STPs since the feeding sludge to the dewatering processes of the STPs is from the ADs; depending on the performance of the ADs, different pharmaceuticals can be more degraded. Unfortunately, we have not investigated carefully looked into the sludge treatment processes of the STPs. Probably, the future study should also emphasize the sludge treatment processes for better understanding of the fate of pharmaceuticals in the reject water.

#### 3.1.3. Concentrations of Pharmaceuticals in Suspended Solids

Twenty of the 27 types of substances were detected at least once in the SS in the influent. Caffeine and naproxen showed 100% detection frequency, and 13 types of substances including acetylsalicylic acid, clarithromycin, and roxithromycin showed at least 50% detection frequency. In contrast, the concentrations of carbamazepine, oxolinic acid, propranolol, sildenafil, sulfamethazine, sulfamethoxazole, and trimethoprim were below the LOQ. Acetylsalicylic acid showed the highest concentration at 641 ng/g. The other substances in decreasing order of their concentrations were ibuprofen (410 ng/g), caffeine (280 ng/g), acetaminophen (220 ng/g), atenolol (210 ng/g), and clarithromycin (150 ng/g) (Figure 1d). Ashfaq et al. (2017) reported mean concentrations of ketoprofen, ciprofloxacin, and ofloxacin in SS of 59 ng/g, 390 ng/g, and 4700 ng/g, respectively [13], which were 10–100 times higher than the results of this study. The concentrations of some substances, such as diphenhydramine, sulfadimethoxine, erythromycin, testosterone, and gemfibrozil, were higher than the LOQ but less than 20 ng/g.

#### 3.1.4. Concentrations of Pharmaceuticals in Sludge

In the activated sludge collected from the aerobic tank, 25 types of substances were detected at least once and 16 types of substances showed at least 50% detection frequency. Only ofloxacin showed a 100% detection rate among the target substances. The concentration of ibuprofen was the highest at 1300 ng/g, followed by cefradine (1100 ng/g), acetylsalicylic acid (890 ng/g), ofloxacin (790 ng/g), clarithromycin (440 ng/g), and cimetidine (430 ng/g) (Figure 1e). Other researchers reported concentrations of 100–4000 ng/g for ibuprofen and 1000–5300 ng/g for ofloxacin in the sludge of STPs, which are higher than those reported in this study [30,31,32]. However, the concentrations of testosterone, gemfibrozil, and erythromycin were low at 20 ng/g. In the waste sludge, 25 types of substances were detected at least once and 19 showed at least 50% detection frequency. In addition, ofloxacin, caffeine, cimetidine, ciprofloxacin, and diclofenac were detected in every sample of the activated sludge. The mean concentrations of ibuprofen, acetylsalicylic acid, ciprofloxacin, ofloxacin, and cimetidine adsorbed on the waste sludge were 1300 ng/g, 1000 ng/g, 900 ng/g, 870 ng/g, and 820 ng/g, respectively (Figure 1f). In particular, ciprofloxacin and ofloxacin showed maximum concentrations of 5100 ng/g and 4800 ng/g, respectively, in the waste sludge. The concentrations varied greatly with the sludge characteristics of the STP. Tran et al. (2018) also reported that the detected concentrations of pharmaceuticals adsorbed on activated and waste sludge of STPs vary widely from the LOQ to over 10 µg/g owing to the usage patterns and physicochemical properties of the individual substances [27]. The pharmaceutical concentrations detected in activated sludge and waste sludge were similar at 15–1300 ng/g, but there was no statistically significant difference between the adsorbed concentrations on the two types of sludge (*p* > 0.05).

### 3.2. Mass Loads and Distribution of Pharmaceuticals in Sewage Treatment Plants

#### 3.2.1. Relative Distributions of Each Therapeutic Class of Pharmaceuticals in Sewage Treatment Plants

**Figure 2:**
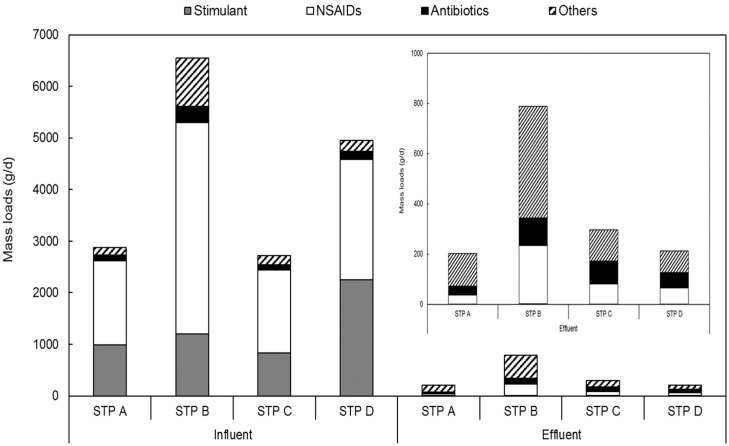
Mass loads and relative distributions of each therapeutic class of pharmaceuticals in the (**a**) influent and (**b**) effluent; sewage treatment plant (STP) A: a modified Ludzack-Ettinger (MLE) followed by an membrane bioreactor (MBR), STP B: an MLE followed by an SBR, STP C: a modified A2O with the anoxic tank divided into two, and STP D: a modified 5-stage Bardenpho with the third stage divided into a media reactor and an aerobic suspended sludge.

The pharmaceuticals detected in the target STPs were classified into four types, namely stimulants, NSAIDs, antibiotics, and others, in order to assess their relative distributions and mass loads by use (Figure 2). The mass loads of NSAIDs in the influent were 1624 g/d for STP A, 4109 g/d for STP B, 1604 g/d for STP C, and 2329 g/d for STP D. The relative distributions of the four types of pharmaceuticals mentioned above were 56%, 63%, 59%, and 47%, respectively, with NSAIDs showing a much higher relative distribution than the other types. STP B had a higher mass load than the other STPs, as its flow rate was 1.7–1.9 times higher. The relative distribution of STP C, which had the lowest NSAID mass load, was the second highest after STP B. We observed no relationship between mass load and relative distribution. A previous study also investigated the relative distributions of 15 types of pharmaceuticals in WWTPs; six types of analgesics and NSAIDs showed the highest relative distributions (70% or more) [33]. The mass load of NSAIDs among the effluents was the highest at 231 g/d in STP B, and ranged from 36–231 g/d in the four STPs. In the case of NSAIDs, the effluent mass load decreased by at least 94% when compared with the influent mass load in the four STPs; however, as the influent mass load was much higher for NSAIDs than for other substance types, their relative distribution in the effluent was also high.

For caffeine (classified as a stimulant), the influent mass load was 990 g/d for STP A, 1197 g/d for STP B, 833 g/d for STP C, and 2253 g/d for STP D with the relative distribution ranging from 18–45%, which was the second highest after NSAIDs. The effluent mass load for stimulants decreased by at least 99% in comparison with the influent mass load in the four STPs, and their relative distribution in the effluent was less than 1%. Gao et al. (2016) also reported that 3848 g/d of caffeine flowed into the WWTP, more than 99% of which was present in the liquid phase [34]. In the biological treatment process, the mass load of the liquid phase was almost completely removed and reduced to 2 g/d in the secondary effluent. We observed the influent mass load of antibiotics as 121 g/d for STP A, 303 g/d for STP B, 107 g/d for STP C, and 160 g/d for STP D. The relative distributions in the four plants was low at 3%–5%, but the effluent mass load was 35 g/d for STP A, 111 g/d for STP B, 90 g/d for STP C, and 62 g/d for STP D with a relative distribution of 14%–30%. Wang et al. (2018) investigated the relative distributions of antibiotics in seven WWTPs in China, and reported a mean relative distribution of 34% in the influent and 28% in the effluent [20]. While this is higher than the influent relative distribution of antibiotics investigated in this study, the effluent relative distributions in both studies are similar. For antibiotics, the effluent mass load decreased in comparison with the influent mass load depending on the STP (71% in STP A and 15% in STP C). Previous studies reported low removal efficiencies of antibiotics in STPs [12,35]. The influent mass load of antiarrhythmics, antihistamines, and anticonvulsants was the highest in STP B at 941 g/d, and ranged from 141–941 g/d in the four STPs. The influent relative distribution of other substances ranged from 5%–14%, which was lower than that of other substances; however, the effluent relative distribution was the highest at 40%–64%. Thiebault et al. (2017) reported the influent relative distributions of beta blockers and psychotropic drugs as 16% and 2%, respectively, although their corresponding effluent relative distributions increased to 56% and 15% [33]; these results are similar to the relative distributions of substances classified as “others” in this study. The effluent mass load in comparison with the influent mass load was reduced by 10% at STP A, 53% at STP B, 31% at STP C, and 60% at STP D. The decrease in the mass loads was the highest in STP D and the lowest in STP A; overall, there was not much reduction in the mass loads at the STPs. Similar to antibiotics, the treatment level differed across the STPs.

#### 3.2.2. Mass Loads of Pharmaceuticals in Each Treatment Process

Table 2 shows the mass loads of pharmaceuticals in the various unit processes of the STPs. Tables S5–S8 show the mass loads and distribution characteristics at each STP. The mass loads (liquid + solid phase) of the pharmaceuticals flowing into the STPs were 2876 g/d for STP A, 6550 g/d for STP B, 2725 g/d for STP C, and 4953 g/d for STP D. Among these, the mass loads of the solid phase were 16 g/d for STP A, 21 g/d for STP B, 10 g/d for STP C, and 30 g/d for STP D, thereby showing that the majority of the substances were contained in the liquid phase. Wang et al. (2018) reported 6570 g/d and 1930 g/d of liquid and solid phase mass loads, respectively, in the influent [20], which are higher than the influent solid phase mass loads found in this study. This is because many highly adsorbable antibiotics were included in their target substances. In contrast, they reported an influent solid phase mass load of less than 10 g/d for NSAIDs, which is similar to the mass loads of the solid phase and relative distributions of liquid and solid phases in the influent observed in this study. As mentioned in Section 3.2.1, NSAIDs had the highest mass loads in the influent; among them, the mass load of acetaminophen was highest at 1144–3066 g/d, followed by that of acetylsalicylic acid (130–612 g/d), ibuprofen (60–590 g/d), and naproxen (86–221 g/d). Among the substances other than NSAIDs, caffeine (832–2242 g/d), iopromide used as a contrast agent (30–604 g/d), and the antihistamine cimetidine (95–287 g/d) showed high mass loads. Previous studies have reported high mass loads for compounds such as acetaminophen (293–14,274 g/d), caffeine (190–2824 g/d), ibuprofen (162 g/d), and naproxen (76–1301 g/d) in the influents of STPs in the USA, New Zealand, and South Korea, which is consistent with the results from this study [14,36,37].

The mass loads of the activated sludge and return sludge were 2226 g/d and 4811 g/d in STP A, 1720 g/d and 1203 g/d in STP B, 959 g/d and 6006 g/d in STP C, and 675 g/d and 1279 g/d in STP D, respectively. Ibuprofen (activated sludge: 85–869 g/d; return sludge: 211–1950 g/d), cimetidine (85–344 g/d; 188–552 g/d), acetylsalicylic acid (110–254 g/d; 134–1207 g/d), and ofloxacin (29–300 g/d; 48–399 g/d) showed high mass loads. With the exception of STP B, the mass loads of the return sludge were higher than those of the activated sludge; the return rate of STP B was 25% (0.25Q), which was at least 2 times lower than that of the other treatment plants. In the activated sludge, substances classified as “others,” such as cimetidine, carbamazepine, and iopromide, were distributed at a higher rate in the liquid phase than in the solid phase except for in STP A, while the other types of substances were contained at a much higher rate in the solid phase. At least 98% of the stimulants and 93% of the antibiotics were contained in the solid phase of the return sludge in the four STPs. All types of substances were distributed at a much higher rate in the solid phase than in the liquid phase. Ofloxacin had a mass load of 620 g/d and ciprofloxacin had a mass load of 47 g/d in samples taken between the anoxic and aerobic tanks, and at least 99% of both substances was found in the solid phase [17]. This was higher than the solid phase ratio of the antibiotics investigated in the activated sludge of the four STPs in this study (65%–96%), and was similar to the ratio determined in the return sludge (93%–97%). Fluoroquinolone substances such as ofloxacin and ciprofloxacin have a low octanol-water partition coefficient (log K_ow_), which indicates low hydrophobicity. However, they reportedly have zwitterionic properties and high adsorption owing to electrostatic interactions [38]. Macrolide antibiotics, such as clarithromycin and roxithromycin, also showed high solid phase ratios in the activated sludge and return sludge. These substances have high log K_ow_ values, thereby indicating that they are more hydrophobic than the other substances. Tran et al. (2016) reported that adsorption occurs between the positively charged dimethylamino group and the negatively charged sludge surface [39]. The waste sludge mass load was 58 g/d in STP A, 48 g/d in STP B, 25 g/d in STP C, and 19 g/d in STP D, with 80% or more contained in the solid phase. The waste sludge mass load to influent mass load was 2% or less in all four STPs, with a small amount removed through each STP process. This particularly requires attention because substances such as antibiotics may pose environmental risks to natural organisms and cause antibiotic resistance when released from sludge into aquatic ecosystems [40,41].

#### 3.2.3. Residual Proportion of Pharmaceuticals in Each Treatment Process

**Figure 3:**
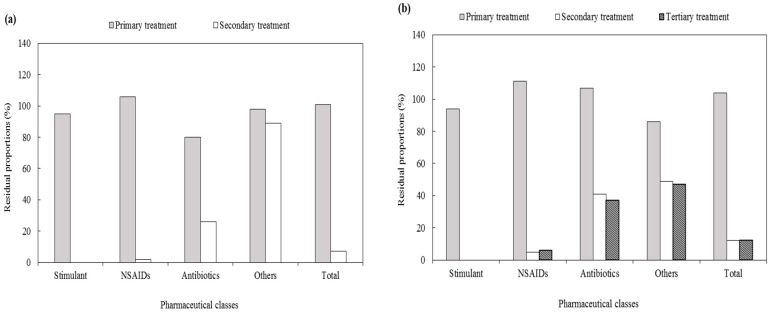

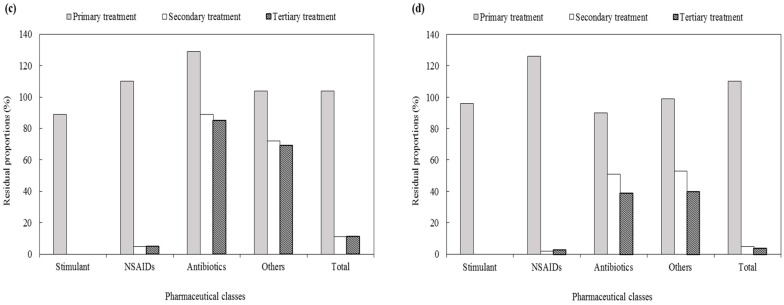
Residual proportions of pharmaceutical classes in each treatment process of the STPs. (**a**) STP A, (**b**) STP B, (**c**) STP C, (**d**) STP D.

Figure 3 shows the residual proportions of pharmaceuticals in the various unit processes of the STPs. In each STP, the total mass load of the primary effluent increased by 1%–10% compared with the total influent mass load; similar to the characteristics of the influent, the mass loads of the solid phase were insignificant. The mass loads of NSAIDs in the primary effluent increased by 6%–26%. Ibuprofen had a maximum mass load of 893 g/d at STP D whereas that of acetaminophen was 3567 g/d at STP B; in each of the four STPs, the mean increase was at least 27% and 16%, respectively. The mass load of antibiotics decreased by 20% in STP A and 10% in STP D through the primary treatment, but increased by 7% in STP B and 29% in STP C. Among the antibiotics that showed an increase in the mass load in the primary effluent, the mass load of cefradine increased from 170 g/d to 186 g/d and that of roxithromycin increased from 21 g/d to 25 g/d in STP B; the mass load of cefradine increased from 38 g/d to 62 g/d and that of clarithromycin increased from 35 g/d to 38 g/d in STP C. Similarly, Ashfaq et al. (2017) investigated the behavior of 49 types of pharmaceuticals and personal care products in STPs using the A2O process, and reported that the mass loads of certain substances such as NSAIDs and antibiotics were higher in the effluent passing through the screens and rotating grit chamber than in the influent [17]. In contrast, in STP B, iopromide decreased from 606 g/d to 475 g/d and ciprofloxacin decreased from 13 g/d to 10 g/d; in the four STPs, they decreased by 14%–29% and 5%–22% through primary treatment, respectively. The total mass load of the secondary effluent was 193 g/d for STP A, 785 g/d for STP B, 313 g/d for STP C, and 251 g/d for STP D. The residual proportion of the secondary effluent was 7% in STP A, 12% in STP B, 11% STP C, and 5% in STP D, which was a reduction of 88%–95% when compared with the influent mass load. In the MBR process of STP A, SS were completely removed after membrane filtration; as a result, all the pharmaceuticals were present in the liquid phase. Most SS were contained in the liquid phase in the other treatment plants as well. In the case of effluent subject to tertiary treatment, the total mass loads were 789 g/d at STP B, 297 g/d at STP C, and 212 g/d at STP D. The residual proportion of the effluent was 12% in STP B, 11% in STP C, and 4% in STP D. STP A was not considered because the substances were discharged from this plant without further treatment after biological treatment. Despite the low concentration of SS in the effluent, certain pharmaceuticals were found in the solid phase; however, most were distributed in the liquid phase, as was the case for the effluent subject to biological treatment.

### 3.3. Comparative Evaluation of Pharmaceutical Removal Efficiencies of Different Treatment Technologies in Sewage Treatment Plants

#### 3.3.1. Removal of Pharmaceuticals by Biological Treatment Processes

**Figure 4:**
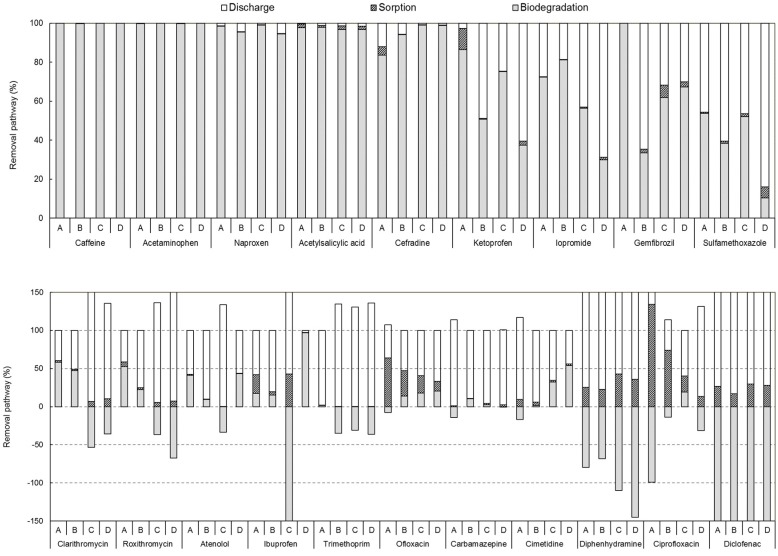
Mass balance of pharmaceuticals in different biological treatment processes in STPs. **A**: MBR, **B**: SBR, **C**: anaerobic–anoxic–oxic (A2O), **D**: moving-bed biofilm reactor (MBBR).

We calculated the mass loads of substances flowing into and discharged from the bioreactor, as well as their mass loads in the waste sludge. Among the 27 types of substances flowing into the bioreactor, we evaluated the removal mechanism by biodegradation and sorption for 20 types (Figure 4). Seven substances with mass loads under 1 g/d (sulfadimethoxine, sulfamethazine, oxolinic acid, erythromycin, sildenafil, testosterone, and propranolol) were excluded from the analysis. Caffeine and acetaminophen, which had high mass loads in the STP influent, were almost completely removed by biodegradation in all treatment processes (≥99%). Naproxen, acetylsalicylic acid, and cefradine were removed by an average of at least 95% by the four treatment processes. Sludge sorption showed a removal rate of less than 2% on average, thereby indicating that biodegradation was the main removal mechanism. Baalbaki et al. (2016) reported similarly high removal efficiencies for these substances; in a municipal WWTP using CAS, naproxen and caffeine were not removed by sludge sorption, but were decreased by 94% and 92%, respectively, by biodegradation [18]. Kim et al. (2014) reported that acetaminophen, caffeine, and naproxen were removed by at least 99% through biological degradation/transformation in a MBR system [42]. Ketoprofen, iopromide, gemfibrozil, and sulfamethoxazole showed removal efficiencies of 40%–97%, 31%–81%, 35%–100%, and 16%–54% in the four treatment processes, thereby indicating that the removal efficiencies varied based on the treatment process. In the MBR process, ketoprofen showed a removal efficiency of 86% through biodegradation and 11% through sludge sorption, which was a total of 97%. This was the highest among the four treatment processes, followed by A2O (75%), SBR (51%), and MBBR (40%) processes. Removal by sludge sorption (2%) was insignificant except for in the MBR process. The removal efficiency of ketoprofen in the MBR process was similar to that reported by Gurung et al. (2019), who observed ketoprofen removal efficiencies of 90% through biodegradation and 8% through sludge sorption in pilot-scale MBRs [43]. In the WWTP, the pharmaceutical removal mechanisms using the A2O process achieved approximately 60% removal efficiency of ketoprofen through biodegradation, with very little removal through sorption [14]. Gemfibrozil was completely removed through biodegradation in the MBR process and was not affected by sludge sorption. However, with a high log K_ow_ value of 4.8, more gemfibrozil tended to be adsorbed and removed in the sludge than other substances, even though it had a low removal efficiency overall. Unlike the abovementioned substances, iopromide had the highest removal efficiency in the SBR process (81%). As a hospital and medical complex were located near STP B, which used the SBR process, the influent mass load of iopromide (contrast agent) was at least 10 times higher than that in the other treatment plants, thereby resulting in higher removal efficiency. The removal of iopromide by sludge sorption was insignificant. It was mainly removed by biodegradation, the results of which were consistent with those reported by Joss et al. (2005) [44]. An evaluation of the removal mechanism of iopromide in the CAS and MBR processes showed that it was not removed by sludge sorption, and that the biodegradation removal ranged from 30% to 95%.

The removal efficiencies of clarithromycin and roxithromycin ranged from −47% to 60% and −60% to 59%, respectively, according to the treatment process. The two compounds were relatively hydrophobic and showed a higher tendency to be removed by sludge sorption. In the A2O and MBBR processes, the mass loads discharged from the bioreactor were higher than the mass loads flowing into the bioreactor, thereby resulting in negative removal efficiencies. Similar to the results in this study, the removal efficiency of clarithromycin was −340% to 15% in three WWTPs using activated sludge and BNR processes, thereby indicating that the substances were discharged without treatment in some WWTPs [19]. Lin et al. (2018) evaluated the removal mechanism of roxithromycin in a municipal WWTP using the A2O process; although it was removed to some extent by sludge sorption (approximately 5%), the mass load contained in the effluent was higher than the influent load, which indicated inadequate removal efficiency of the treatment process [32]. Similarly, roxithromycin showed a removal efficiency of −20% to 60% by biodegradation and sludge sorption, and the removal efficiency differed according to the treatment process [44]. For the fluoroquinolones ofloxacin and ciprofloxacin, the contribution of sludge sorption was 13%–64% and 13%–134%, respectively, which was higher than that of other substances. Studies have reported that sorption accounts for much higher removal of these substances than biodegradation because of the large amount transferred from the liquid phase to the solid phase in the activated sludge and waste sludge [20]. Relatively high adsorption properties were also observed for diphenhydramine and diclofenac. The contributions of sludge sorption for these two compounds were 23%–43% and 17%–30%, respectively; however, the amount of discharged substances was highest among all treatment processes, thereby indicating a negative removal efficiency. Diclofenac and diphenhydramine were found to be recalcitrant in all biological treatment processes. Likewise, the removal efficiencies for trimethoprim and carbamazepine were −36% to 2% and −13% to 11%, respectively, in the four treatment processes, and the overall removal efficiency was low or negative. The removal efficiencies for atenolol and cimetidine ranged from −33% to 44% and from −8% to 56%, respectively. Although atenolol and cimetidine showed negative removal efficiencies in some biological treatment processes, they were hardly or moderately removed by biodegradation. Similar to the results of this study, some compounds such as carbamazepine, diclofenac, ciprofloxacin, clarithromycin, roxithromycin, and trimethoprim showed negative removal efficiencies in certain biological treatment processes [17,19,32,42]. This may be attributed to retransformation of the metabolites or conjugate forms into their original forms during biological treatment; this retransformation eventually increases the concentrations of the original compounds in the effluent [11,45]. Previous studies have reported that the fluctuation in concentration due to grab sampling [46] and the desorption of adsorbed compounds in the solid phase [47] could cause negative removal efficiency. However, we can exclude these causes because this study analyzed composite samples of both the liquid phase and solid phase for each unit operation. In the case of highly hydrophobic compounds, a relatively wider recovery range could be obtained for the solid phase samples, which might result in some errors in measurements. The measurement errors might tend to be greater at low levels, particularly as the LOQ is approached. Although consistent recovery rates are obviously preferred, in the case of the simultaneous analysis of multiple pharmaceuticals, it is practically difficult or impossible to achieve the acceptance criteria for all compounds. Alternatively, the intra-day repeatability and the inter-day reproducibility were assessed to verify the measured data. They showed the acceptable range of less than 20%. Also, the fluctuation in recovery rates of the solid samples might not significantly affect the final result associated with the negative removal efficiencies since the portions of target compounds distributed in the solid phase in the primary effluent (0.3%–0.8%) and secondary effluent (0.1%–4.4%) of each STP was considerably lower than those in the liquid phase. Regarding substances that were not efficiently removed in the biological treatment process, it is necessary to evaluate the behavior of the substances produced through metabolism in the treatment process in future studies to understand them more accurately.

**Figure 5:**
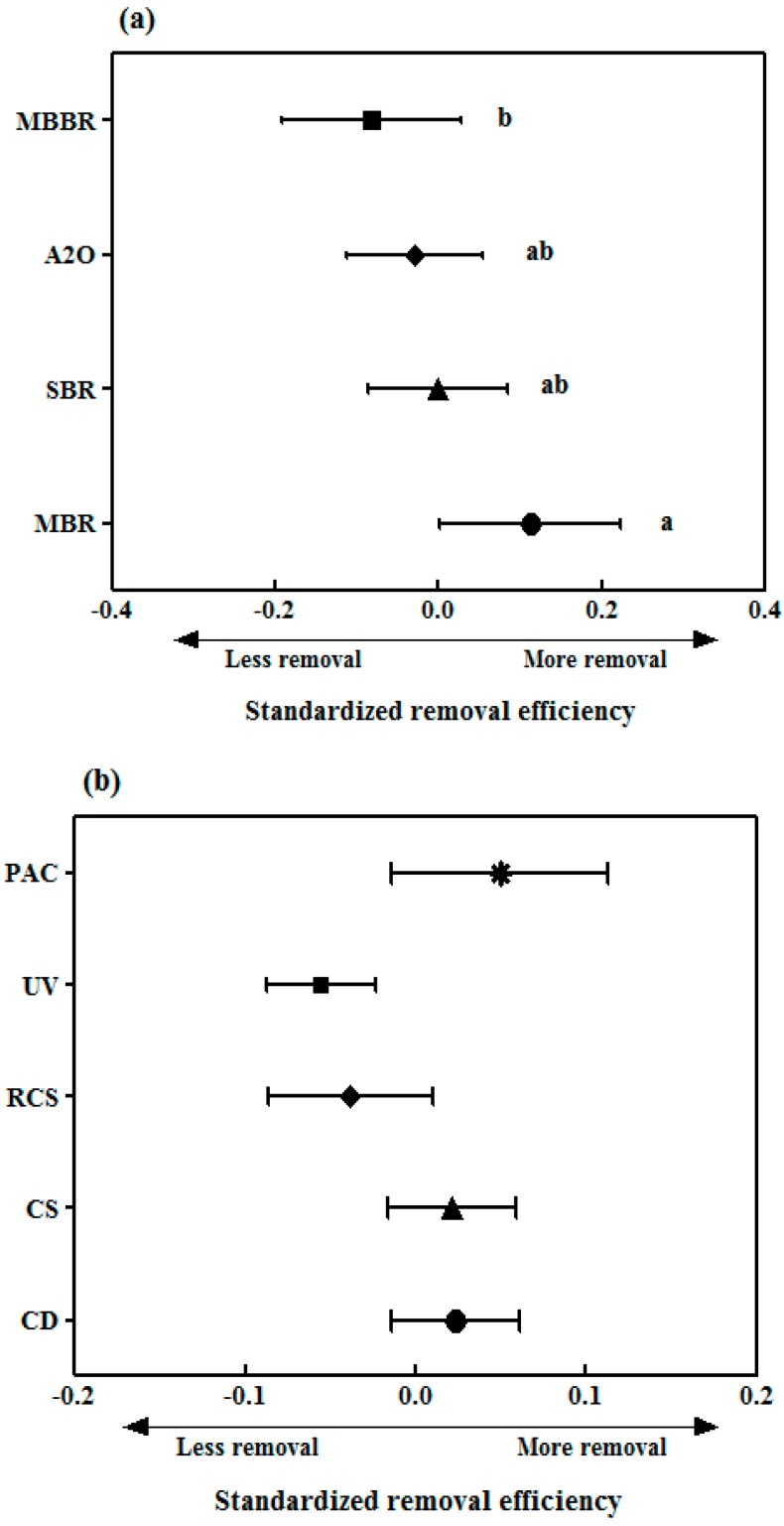
Comparison of standardized removal efficiencies (SREs) (mean ± 95% CI) of pharmaceuticals in different treatment processes. (**a**) Biological treatment processes and (**b**) tertiary treatment processes. A significant difference (*p* < 0.05) between SREs of different treatment processes is represented by alphabets, based on ANOVA with Tukey post-hoc test. MBR: membrane bioreactor, SBR: sequencing batch reactor, A2O: anaerobic–anoxic–oxic, MBBR: moving-bed biofilm reactor, CD: coagulation/disk filter, CS: coagulation/sedimentation, RCS: rapid coagulation-sedimentation, UV: ultraviolet, and PAC: powdered activated carbon.

The meta-analysis showed that the values of SRE of the four treatment processes were in the order of MBR > SBR > A2O > MBBR (Table S9); only the difference between the MBR process with the highest removal efficiency and the MBBR process with the lowest removal efficiency was statistically significant (*p* < 0.05; ANOVA) (Figure 5a). The operating conditions of each treatment process must be examined to explain the high SRE values in the MBR process. The operating parameters, such as SRT, mixed liquor suspended solids (MLSS) concentration, and food-to-microorganism (F/M) ratio, differed for each treatment process (Table S1). The SRT was the longest at 21.5 d for the MBR process, and the MLSS concentration was 2–3 times higher than that in the other processes. Previous studies have reported that long SRTs increase the activity and diversity of microorganisms in STPs and improve the biodegradability of many pharmaceuticals [48,49]. Long SRTs in the MBR proliferate autotrophic slow-growing microorganisms, which cometabolize pharmaceuticals through non-specific enzymes [50,51]. In addition, the F/M ratio in the MBR process was 0.05 kg BOD/kg MLSS d; this was four times lower than that of the MBBR process, which had the lowest SRE. MBR processes with a low F/M ratio are known to improve the removal of pharmaceuticals by making the microorganisms metabolize substances that are difficult to degrade owing to relatively low influent substrate concentrations [52,53]. In some studies, a biofilm was formed on the surface of the MBBR, thereby enhancing the degradation of the pharmaceuticals by microorganisms. Therefore, this technology can be considered more effective than CAS and MBR processes in removing compounds such as X-ray contrast media, atenolol, diclofenac, naproxen, and gemfibrozil [54,55]. However, the results of the MBBR process in this study appeared to differ from those of previous studies because the SRT was the shortest at 11.8 d and the F/M ratio was the highest at 0.21 kg BOD/kg MLSS d among the four treatment processes.

The removal of pharmaceuticals during the biological treatment process depends on not only the operating conditions but also the diversity and function of microbial communities. The changes in operating conditions are considerably related to microbial population dynamics in biological treatment processes. The shifts of microbial communities strongly impact on the removal of pharmaceuticals, as reported by recent studies [56,57]. This may be one of the reasons why the order of SREs was different in individual treatment processes. For instance, Gallardo-Altamirano et al. (2019) compared removal efficiencies of 19 pharmaceuticals under two experimental phases (different MLSS concentration and F/M ratio), reporting that the removal efficiencies of some pharmaceuticals such as clarithromycin, gemfibrozil, and atenolol increased twofold at high MLSS concentration and low F/M ratio [58]. The degree of microbial diversity and pharmaceutical removal are influenced by operational parameters, such as SRT, redox condition, and carbon supply [57,59]. Stadler et al. (2018) conducted batch experiments for the pharmaceutical biotransformation and observed that metabolic genes, such as dehydrogenases, amidases, and monooxygenases, were positively associated with the extent of pharmaceutical biotransformation [56]. In order to better understand the removal characteristics of pharmaceuticals in biological treatment processes, future work should consider the effects of operating conditions on variations in microbial communities and possible relations between these microbial communities and the removal of pharmaceuticals.

#### 3.3.2. Removal of Pharmaceuticals in Tertiary Treatment Processes

We evaluated the performance of the tertiary treatment process for 16 types of substances whose influent mass loads were greater than 1 g/d and whose removal efficiencies were not significantly negative in the tertiary treatment. In South Korea, the water quality standard for total phosphorus in the effluents from public STPs was strengthened from 2.0 mg/L by 0.2 mg/L to 0.5 mg/L in 2012 to suppress the occurrence of algae in rivers and lakes. Of the 604 STPs with a treatment capacity of more than 500 m^3^/d, 62% are equipped to remove total phosphorus, among which disk filters and filtration systems account for 53% and sedimentation/flotation account for 39%. Therefore, we compared the pharmaceutical removal efficiencies in total phosphorus treatments based on coagulation, such as coagulation/disk filter (CD), coagulation/sedimentation (CS), and rapid coagulation-sedimentation (RCS) processes, in addition to comparing the pharmaceutical removal efficiencies with the removal efficiencies of five types of tertiary treatment processes, including UV and PAC treatments, for disinfection and reuse (Figure 5b). A meta-analysis showed that the removal efficiencies of pharmaceuticals derived from the five tertiary treatment processes were not statistically significant (*p* > 0.05; ANOVA). In the tertiary treatment processes, the SRE decreased in the order of PAC > CD > CS > RCS > UV. The SRE values were similar for the CD and CS processes in the three total phosphorus treatment plants, whereas it was lower for the RCS process. In coagulation plants used for the removal of organic substances and total phosphorus, the removal of pharmaceuticals has not been observed to have a significant effect [60], but compounds with a log K_ow_ value of 4 or higher have been reported to improve the removal efficiency [61]. Gemfibrozil, namely the only compound for which the log K_ow_ value was higher than 4, did not improve the removal efficiencies of the three total phosphorus treatment plants despite being hydrophobic. A similar removal efficiency was observed for CS even after passing through two disk filters (fiber disk type) after coagulation treatment in a CD, thereby indicating that filtration through disk filters did not have a significant influence on the removal of pharmaceuticals. This was because the molecular size of the pharmaceuticals was much smaller than the pore size of the disc filters, thereby making disc filters ineffective at removing these compounds [14]. Similarly, UV treatment did not show any significant effect on the removal of pharmaceuticals. This agreed with the study by Yang et al. (2014), who mentioned that using UV irradiation for disinfection in a WWTP was not effective in removing most trace contaminants [62].

The removal efficiency of PAC was the highest among the five types of tertiary treatment processes evaluated. The removal efficiencies for compounds with relatively high hydrophobicity (log K_ow_ value of 3.1–4.8), such as gemfibrozil, ibuprofen, and ketoprofen, increased by 18%–63% through the PAC treatment. On the other hand, the removal efficiencies of atenolol, cimetidine, and trimethoprim, which have low hydrophobicity (log K_ow_ value of 1 or less), increased by 42%, 35%, and 20%, respectively. Similarly, Guillossou et al. (2019) compared the effluent concentrations of a WWTP and a pilot-scale activated carbon treatment [63]. The removal efficiencies of atenolol, ketoprofen, and trimethoprim increased by 10%–25%; they explained that the physicochemical properties of the target compounds, such as size, charge, polarity, and functional group, were not significantly related to the removal efficiency for trace contaminants. This was because although the main treatment mechanism of PAC is adsorption, this single factor alone cannot explain the increase in the removal efficiency; rather, it is influenced by complex physicochemical interactions between the target substance, activated carbon, and dissolved organic substances [64,65]. In order to improve the removal efficiency for the hardly removable compounds, various adsorbents are being developed and applied. For example, porous materials synthesized with nano-carbon tubes or biochar have been applied to remove trace pharmaceuticals and dues from water [66,67]; the results were very promising, so it was expected that pharmaceutical-free water can be discharged if these adsorption technologies are properly designed and applied to remove residual pharmaceuticals after a biological process in an STP. However, still trace pharmaceuticals could be detected even after an adsorption process is applied after a biological one. Therefore, a more integrated system combing adsorption and oxidation processes might be desirable as proposed by Du et al. [68] or Sharma et al. [69].

#### 3.3.3. Practical Suggestions to Enhance the Pharmaceutical Removal in Sewage Treatment Plants

Since it is presumed that pharmaceuticals should, if discharged, negatively affect the water environment, they need to be degraded in a STP. As presented above, however, it has been turned out that pharmaceuticals could not be completely removed in none of the STPs where sampling was performed. Moreover, it is difficult to conclude which compounds are efficiently removed by which STP. Nonetheless, our results showed that two processes (i.e., MBR in the biological treatment process and PAC in the tertiary treatment process) could more effectively treat pharmaceuticals than other treatment processes. Several studies have demonstrated that MBR and PAC can be cost-competitive when the effluent quality level, footprint, and operational costs are collectively considered. For example, the average footprint of an MBR (0.9 m^2^∙m^−3^∙d) is smaller than those of conventional activated sludge processes (1.2–1.6 m^2^∙m^−3^∙d) [70]. Activated carbon adsorption is a preferred method for micropollutants removal in pioneering European countries owing to its acceptable removal performance, technical feasibility, and cost-effectiveness [71]. In particular, Switzerland has enacted and implemented laws to implement advanced treatment technologies, such as PAC or ozone processes, in 100 out of 700 STPs (equivalent to approximately 50% of the total sewage treatment capacity of the nation) in order to prevent trace contaminants from being discharged to aquatic ecosystems [72]. Thus, we believe that if both MBR and PAC are implemented in a STP pharmaceuticals in sewage can relatively effectively controlled, thereby minimizing the amount of pharmaceuticals discharged into water bodies. We expect such a technology to serve as a favorable option for ensuring the sustainability of our water environment.

## 4. Conclusions

In this study, the behavior and distribution of 27 pharmaceuticals for liquid and solid phase samples in four STPs were investigated. A comparative evaluation on removal efficiencies of representative treatment processes used in South Korea was also performed based on the mass balance and meta-analysis.

Most substances were present in the liquid phase in the four STPs, and the proportion distributed in the solid phase in the influent was less than 2%. After biological treatment, only 5%–12% of the influent mass loads remained, and the mass loads discharged owing to waste sludge in all processes were less than 2%.

Caffeine, acetaminophen, naproxen, acetylsalicylic acid, and cefradine were removed to a great extent by biodegradation in all the STPs. The removal efficiencies of ketoprofen, iopromide, gemfibrozil, and sulfamethoxazole varied depending on the contributions of biodegradation and sludge sorption.

A comparison of the SREs of the pharmaceuticals in the four types of biological treatments showed that the SRE decreased in the order of MBR > SBR > A2O > MBBR. The difference between the removal efficiencies of the MBR and MBBR processes was statistically significant.

Among five types of tertiary treatment processes, PAC showed the most efficient performance, with an increase of 18%–63% in the removal efficiency for compounds including gemfibrozil, ibuprofen, ketoprofen, atenolol, cimetidine, and trimethoprim.

Overall, our findings provide an overview of the elimination and behavior of pharmaceuticals in different unit processes of STPs in South Korea. We believe this paper will provide useful information to those who design or operate STPs regarding occurrence and fate of pharmaceuticals in sewage.

## Figures and Tables

**Table 1:** Physicochemical properties of target compounds.

Pharmaceuticals	Molecular Formula	Molecular Weight (g/mol)	Octanol-Water Partition Coefficient (Log K_ow_)	Acid Dissociation Constant (p*K*a)	Water Solubility (mg/mL)
**Analgesics/Non-steroidal anti-inflammatory drugs (NSAIDs)**					
Acetaminophen	C_8_H_9_NO_2_	151.2	0.5	9.4	30.4
Acetylsalicylic acid	C_9_H_8_O_4_	180.2	1.2	3.5	4.6
Diclofenac	C_14_H_11_C_l2_NO_2_	296.2	3.9	4.2	4.5 × 10^−3^
Ibuprofen	C_13_H_18_O_2_	206.3	3.6	4.9	4.1 × 10^−2^
Ketoprofen	C_16_H_14_O_3_	254.3	3.1	4.5	0.1
Naproxen	C_14_H_14_O_3_	230.3	3.2	4.2	0.1
**Antibiotics**					
Cefradine	C_16_H_19_N_3_O_4_S	349.4	−0.3	2.6/7.3	2.8
Ciprofloxacin	C_17_H_18_FN_3_O_3_	331.4	0.3	6.1/8.7	11.5
Clarithromycin	C_38_H_69_NO_13_	748	3.2	9	3.4 × 10^−4^
Erythromycin	C_37_H_67_NO_13_	734	3.1	8.9	5.2 × 10^−4^
Ofloxacin	C_18_H_20_FN_3_O_4_	361.4	−0.4	6.3/7.9	28.3
Oxolinic acid	C_13_H_11_NO_5_	261.2	0.9	6.9	8
Roxithromycin	C_41_H_76_N_2_O_15_	837.1	2.8	9.2	1.9×10^−5^
Sulfadimethoxine	C_12_H_14_N_4_O_4_S	310.3	1.6	2.1/6.1	0.4
Sulfamethazine	C_12_H_14_N_4_O_2_S	278.3	0.6	2.1/7.5	11.3
Sulfamethoxazole	C_10_H_11_N_3_O_3_S	253.3	0.9	1.6/5.7	3.9
Trimethoprim	C_14_H_18_N_4_O_3_	290.3	0.9	7.1	2.3
**Antiarrhythmic agents**					
Atenolol	C_14_H_22_N_2_O_3_	266.3	0.2	9.6	0.7
Propranolol	C_16_H_21_NO_2_	259.4	0.7	9.4	0.2
**Antihistamines**					
Cimetidine	C_10_H_16_N_6_S	252.3	0.4	6.8	10.5
Diphenhydramine	C_17_H_21_NO	255.4	3.3	9	0.4
**Hormone**					
Testosterone	C_19_H2_8_O_2_	288.4	3.3	-	0.1
**Stimulant**					
Caffeine	C_8_H_10_N_4_O_2_	194.2	−0.1	14	2.6
**Others**					
Carbamazepine	C_15_H_12_N_2_O	236.3	2.5	13.9	1.8 × 10^−2^
Gemfibrozil	C_15_H_22_O_3_	250.3	4.8	4.5	5.0 × 10^−3^
Iopromide	C_18_H_24_I_3_N_3_O_8_	791.1	−2.1	10.6	2.4 × 10^−2^
Sildenafil	C_22_H_30_N_6_O_4_S	474.6	2.8	5.9	3.5

**Table 2:** Mass loads of each therapeutic class of pharmaceuticals in different unit processes of STPs. L: mass loads of pharmaceuticals in the liquid phase, S: mass loads of pharmaceuticals in the solid phase, T: total mass loads of pharmaceuticals.

STPs	Class	Influent			Primary Effluent			Secondary Effluent			Tertiary Effluent			Activated Sludge			Return Sludge			Waste Sludge		
		L	S	T	L	S	T	L	S	T	L	S	T	L	S	T	L	S	T	L	S	T
A	Stimulant	989	1	990	941	4	945	0	0	0	-	-	-	0	21	22	1	65	66	0	0	0
	NSAIDs	1613	11	1624	1697	17	1713	35	0	35	-	-	-	120	1041	1161	102	2883	2985	2	16	18
	Antibiotics	118	2	121	94	2	96	31	0	31	-	-	-	22	610	632	61	990	1051	1	27	28
	Others	140	1	141	138	1	138	127	0	127	-	-	-	154	257	411	106	602	709	2	9	11
	**Total**	**2860**	**16**	**2876**	**2869**	**24**	**2893**	**193**	**0**	**193**	**-**	**-**	**-**	**297**	**1929**	**2226**	**270**	**4541**	**4811**	**5**	**53**	**58**
B	Stimulant	1196	1	1197	1120	1	1121	1	0	1	1	2	3	0	9	9	0	7	7	0	0	0
	NSAIDs	4096	13	4109	4547	18	4564	191	7	198	218	12	231	301	411	712	81	292	373	3	12	15
	Antibiotics	298	6	303	321	4	325	113	10	123	109	2	111	200	368	568	15	413	427	1	16	17
	Others	939	2	941	804	3	807	458	5	463	443	2	444	328	104	432	112	283	395	4	11	16
	**Total**	**6529**	**21**	**6550**	**6791**	**26**	**6817**	**763**	**23**	**785**	**771**	**18**	**789**	**828**	**892**	**1720**	**208**	**995**	**1203**	**8**	**39**	**48**
C	Stimulant	832	1	833	740	1	741	0	0	0	0	0	0	0	4	4	0	111	111	0	0	0
	NSAIDs	1597	7	1604	1762	6	1768	85	2	87	78	3	81	89	294	383	308	2818	3126	2	11	13
	Antibiotics	105	2	107	136	1	137	94	1	95	89	1	90	75	328	403	110	1417	1527	0	6	6
	Others	181	0	182	188	0	188	130	0	131	125	0	125	89	80	169	363	879	1243	2	4	6
	**Total**	**2716**	**10**	**2725**	**2826**	**8**	**2834**	**309**	**4**	**313**	**292**	**5**	**297**	**253**	**706**	**959**	**782**	**5225**	**6006**	**4**	**22**	**25**
D	Stimulant	2242	11	2253	2159	5	2164	1	0	1	1	0	1	1	15	15	0	29	29	0	0	0
	NSAIDs	2315	15	2329	2914	11	2925	55	2	57	60	3	63	93	204	297	75	423	498	1	6	7
	Antibiotics	156	3	160	143	2	144	73	8	82	58	4	62	58	110	169	19	392	411	0	6	6
	Others	210	1	211	208	0	209	111	0	112	85	0	85	136	57	194	96	245	341	1	4	5
	**Total**	**4923**	**30**	**4953**	**5424**	**18**	**5443**	**240**	**11**	**251**	**204**	**8**	**212**	**289**	**386**	**675**	**190**	**1089**	**1279**	**3**	**16**	**19**

## Supplementary Materials

The following are available online at https://www.mdpi.com/1660-4601/17/3/687/s1, Text S1: Pretreatment of solid samples by the QuEChERS method; Text S2. Online SPE-LC/MS/MS analysis; **Table S1**. Influent characteristics and operational parameters of individual STPs; **Table S2**. Description of primary, secondary, and tertiary treatment processes of target STPs. **Table S3**. Operating parameters of online SPE LC-MS/MS. **Table S4**. Limit of quantification (LOQ), recoveries, and relative standard deviations (RSD) for each pharmaceutical in the liquid and solid phase. **Table** S5. Mass loads and residual proportion of pharmaceuticals in different unit processes of STP A. **Table S6**. Mass loads and residual proportion of pharmaceuticals in different unit processes of STP B; **Table S7**. Mass loads and residual proportion of pharmaceuticals in different unit processes of STP C. **Table S8**. Mass loads and residual proportion of pharmaceuticals in different unit processes of STP D. **Table S9**. Standardized removal efficiencies of pharmaceuticals in biological and tertiary treatment processes; **Figure S1**. Process flow diagrams and sampling sites of target STPs. **Figure S2**. Schematic diagram of the pharmaceutical analysis for liquid and solid samples. **Figure S3**. Ratio of reject water to influent of pharmaceuticals in the studied STPs.
