# Supplementary material for: Distribution and Removal of Pharmaceuticals in Liquid and Solid Phases in the Unit Processes of Sewage Treatment Plants
Source: Int J Environ Res Public Health. 2020 Jan 21;17(3):687. doi: 10.3390/ijerph17030687 (PMC7036819; doi:10.3390/ijerph17030687)
Supplement: Supplementary file 1 [file ijerph-17-00687-s001.pdf]

Supplementary Information

Distribution and Removal of Pharmaceuticals in Liquid and Solid Phases in the Unit processes of Sewage Treatment Plants

Junwon Park[a], Changsoo Kim[a], Youngmin Hong[b], Wonseok Lee[a], Hyenmi Chung[a], Dong-Hwan Jeong[a, *], Hyunook Kim[c, *]

[a] Department of Environmental Infrastructure Research, National Institute of Environmental Research, Ministry of Environment, 42 Hwangyeong-ro, Seo-gu, Incheon, 22689, South Korea

[b] Technical Research Center, Shimadzu Scientific Korea, 145, Gasan digital 1-ro, Geumcheon-gu, Seoul, 08056, South Korea

[c] Department of Environmental Engineering, University of Seoul, 163 Seoulsiripdaero, Dongdaemun-gu, Seoul, 02054, South Korea

* Corresponding author

Dong-Hwan Jeong (E-mail: dwcheong@korea.kr)

Hyunook Kim (E-mail: h_kim@uos.ac.kr)

Text S1. Pretreatment of solid samples by the QuEChERS method

We modified the quick, easy, cheap, effective, rugged, and safe (QuEChERS) method to extract the solid phase samples. The detailed methods were described in our previous study [1]. The QuEChERS was developed by Anastassiades et al. (2003) as a method for analyzing multi-component residual pesticides and antibiotics in fruits and vegetables [2]; it was later recognized by the Association of Analytical Communities (AOAC) and the European Committee for Standardization (CEN) [3,4]. Recently, the applicability of the method has expanded to the analysis of trace contaminants such as antibiotics, insecticides, non-steroidal anti-inflammatory drugs, and metabolites adsorbed on soil, manure, and sewage sludge [5,6,7].

Text S2. Online SPE-LC/MS/MS analysis

Conventional methods such as offline solid phase extraction and liquid-liquid extraction for sample pretreatment were time-consuming, labor intensive, and cost-ineffective, thereby leading to a limited throughput [8]. In recent years, online SPE methods have been widely applied in the analysis of trace contaminants such as pharmaceuticals, hormones, and personal and care product to improve the precision and sensitivity through automation [9,10]. In this regard, quantitative analysis of target pharmaceuticals was performed with an SPE-LC-MS/MS system consisting of LC-MS/MS and an on-line SPE column with a switching device, as described in our previous study [10].

Table S1. Influent characteristics and operational parameters of individual STPs.

Parameters	STPs			
	STP A	STP B	STP C	STP D
**Location**	Urban	Urban	Urban	Urban
**Characteristics of sewage**	Domestic	Domestic	Domestic	Domestic
**Influent characteristics**				
Influent BOD (mg/L)	179.8	116.7	185.9	310.5
Influent DOC (mg/L)	52.1	45.0	41.9	90.0
Influent SS (mg/L)	168.5	126.1	184.6	292.5
Influent T-N (mg/L)	33.0	39.3	45.7	50.9
Influent T-P (mg/L)	4.0	4.0	4.7	5.6
**Operational parameters**				
Influent flow rate (m^3/d)	42,500	80,000	47,000	47,000
Sludge production rate (m^3/d)	583	791	396	406
HRT (hr)	10.8	17.6	9.5	11.6
SRT (d)	21.5	12.3	17.4	11.8
MLSS concentration (mg/L)	7495	2230	3310	2990
F/M ratio (kg BOD/kg MLSS d)	0.05	0.07	0.14	0.21

BOD: biochemical oxygen demand, DOC dissolved oxygen carbon, SS: suspended solids, T-N: total nitrogen, T-P: total phosphorous, HRT: hydraulic retention time, SRT: sludge retention time, MLSS: mixed liquor suspended solids, F/M: food-to-microorganism.

Table S2. Description of primary, secondary, and tertiary treatment processes of target STPs.

	Primary treatment	Secondary treatment	Tertiary treatment
	Physical separation of solids and greases from the sewage	Biological removal of pollutants by micro-organisms	Disinfection and removal of nutrients and non-biodegradable pollutants
STP A	Screen	An., Ax., Aer. (including Membrane)	-
STP B	Screen, Grit chamber	Ax., An., Aer.#1, Aer.#2, SBR	Coagulation, 1st disk filter, 2nd disk filter, Ultraviolet
STP C	Grit chamber, Primary clarifier	An., Ax.#1, Ax.#2, Aer., Second clarifier	Coagulation
STP D	Primary clarifier	An., Ax.#1, Bio-SAC, Aer.#1, Ax.#2, Aer.#2, Second clarifier	Coagulation, Powdered activated carbon

An.: anaerobic tank, Ax.: anoxic tank, Aer.: aerobic tank, SBR: sequencing batch reactor.

Table S3. Operating parameters of online SPE LC-MS/MS.

LC parameters	
Online SPE column	MAYI-ODS (G) (2.0 mm x 10 mm)
HPLC column	ACE 5 C18-PFP (150 mm x 2.1 mm)
Mobile phase A	0.1% formic acid in Water
Mobile phase B	Acetonitrile
Mobile phase C	0.1% formic acid in Water
Mobile phase D	Acetonitrile : Methanol : IPA : Water (1:1:1:1)
Gradient elution	10% B pump (0 – 2.5 min) – 100% B pump (13.0 – 17.0 min) – 10% B pump (17.01–20.0 min)
Flow rate A, B	0.2 mL / min
Oven temp.	40 ℃
Injection volume	300 μL
MS parameters	
Nebulizing gas flow	3 L/min
Drying gas flow	10 L/min
Heating gas flow	10 L/min
DL temp.	250 ℃
IF temp.	350 ℃
Heating Block temp.	400 ℃
Ionization method	Electrospray Ionization (ESI)
Data acquisition	Multiple Reaction Monitoring (MRM) mode

The analytical equipment used included an HPLC (Nexera X2) from Shimadzu Corporation, with the peaks identified and quantified using a mass spectrometer (LCMS-8050, Shimadzu). For the mobile phase solution, 0.1% formic acid (A) and acetonitrile (B) were set to a flow rate of 0.2 mL/min. The composition of the mobile phase was 10% B (0–2.5 min)-100% B (13.0–17.0 min)-10% B (17.1–20.0 min). The injection volume was set to 300 μL. We used ACE 5 C18-PFP (150 mm × 2.1 mm) and MAYI-ODS (G) (2.0 mm × 10 mm) for the HPLC column and online SPE column, respectively.

Table S4. Limit of quantification (LOQ), recoveries, and relative standard deviations (RSD) for each pharmaceutical in the liquid and solid phase.

Pharmaceuticals	Liquid phase (n=7)							Solid phase (n=7)						
	LOQ (ng/L)	Low level (10-100 ng/L)		Middle level (400-4,000 ng/L)		High level (800-8,000 ng/L)		LOQ (ng/g)	Low level (10 ng/g)		Middle level (50 ng/g)		High level (100 ng/g)	
		Recovery (%)	RSD (%)	Recovery (%)	RSD (%)	Recovery (%)	RSD (%)		Recovery (%)	RSD (%)	Recovery (%)	RSD (%)	Recovery (%)	RSD (%)
Acetaminophen	31.6	108.2	4.7	107.0	2.3	104.5	1.3	3.3	88.9	11.9	102.3	7.9	90.5	4.5
Acetylsalicylic acid	13.7	91.5	5.9	100.0	6.0	100.9	2.6	3.5	87.2	12.8	93.0	11.3	92.0	5.0
Atenolol	9.3	93.9	8.3	99.4	4.4	101.6	2.4	2.0	85.5	7.4	103.3	3.0	115.1	8.1
Caffeine	3.7	103.4	9.7	101.5	4.7	103.3	4.2	2.7	101.0	8.4	112.1	5.2	99.1	3.1
Carbamazepine	5.7	105.2	10.4	107.7	2.8	107.1	3.6	1.8	90.9	6.2	96.5	1.9	97.9	1.6
Cefradine	4.4	106.5	14.3	98.9	3.9	100.8	1.3	4.0	71.0	17.9	69.1	6.2	68.7	2.9
Cimetidine	4.4	110.6	3.8	102.9	8.5	108.5	2.4	0.7	65.8	3.3	77.5	3.1	69.1	0.8
Ciprofloxacin	3.3	110.8	10.5	94.4	5.8	99.0	5.6	2.1	61.3	12.6	68.3	6.2	64.0	4.5
Clarithromycin	3.3	97.3	7.0	97.3	5.2	101.7	5.3	1.7	115.9	4.8	119.3	3.7	118.9	4.4
Diclofenac	11.2	103.4	10.0	117.2	8.4	110.5	6.4	1.0	94.9	3.2	105.1	0.7	96.1	1.6
Diphenhydramine	1.0	101.4	4.2	95.8	2.6	97.3	2.5	1.8	70.7	8.0	70.3	1.7	68.1	2.0
Erythromycin	2.9	108.6	7.5	106.5	2.1	91.3	1.6	1.3	96.2	4.3	104.7	1.8	101.7	1.8
Gemfibrozil	2.0	104.5	6.3	105.3	2.3	102.8	2.7	1.3	67.5	6.3	67.2	4.9	67.4	1.4
Ibuprofen	11.5	93.3	7.8	104.3	5.8	104.5	5.8	2.6	111.8	7.5	118.8	7.0	137.2	3.1
Iopromide	6.4	98.9	5.9	106.3	3.4	102.0	4.7	2.6	115.0	7.3	109.6	6.5	99.5	7.8
Ketoprofen	2.4	94.2	9.0	98.2	3.2	99.3	2.9	1.1	77.0	4.7	74.1	2.8	71.7	3.8
Naproxen	3.1	112.3	8.9	107.2	3.8	102.3	2.0	1.4	85.3	5.4	84.9	1.2	82.4	3.9
Ofloxacin	1.4	112.5	5.4	99.6	4.1	98.0	3.1	3.1	101.6	9.8	113.7	3.0	114.0	3.6
Oxolinic acid	5.3	106.2	13.2	101.0	2.3	107.8	3.7	1.6	120.6	4.3	105.7	6.7	96.2	4.2
Propranolol	4.9	102.0	5.8	96.5	3.7	100.5	3.9	1.4	96.7	4.6	101.3	2.5	97.0	1.2
Roxithromycin	2.4	95.4	12.7	94.7	1.4	100.9	2.8	1.3	91.9	4.4	96.2	2.5	100.3	4.5
Sildenafil	7.8	116.8	9.9	93.2	2.0	101.7	3.6	1.8	83.7	6.7	87.4	1.7	90.4	1.3
Sulfadimethoxine	1.4	101.4	2.9	100.9	1.7	99.0	2.8	0.6	68.7	2.7	73.0	1.1	67.4	1.7
Sulfamethazine	7.0	98.4	17.5	103.6	12.8	106.5	4.4	1.2	71.1	5.5	78.2	1.3	70.2	7.1
Sulfamethoxazole	5.2	110.5	10.1	112.9	2.1	98.1	1.1	1.0	61.3	5.4	71.8	1.4	66.3	6.7
Testosterone	1.5	111.2	5.6	109.5	1.1	97.5	1.7	0.9	77.1	3.8	85.5	1.6	86.7	2.0
Trimethoprim	9.1	101.1	10.0	91.0	2.2	106.0	4.6	0.7	72.6	3.2	70.6	2.7	71.6	1.7

Minimum	91.5	2.9	91.0	1.1	91.3	1.1	61.3	2.7	67.2	0.7	64.0	0.8
Maximum	116.8	17.5	117.2	12.8	110.5	6.4	120.6	17.9	119.3	11.3	137.2	8.1
Mean	103.7	8.4	102.0	4.0	102.0	3.3	86.3	6.8	91.1	3.7	88.9	3.5
Median	103.4	8.3	101.0	3.4	101.7	2.9	85.5	5.5	93.0	2.8	90.5	3.1

Table S5. Mass loads and residual proportion of pharmaceuticals in different unit processes of STP A.

STPs	Pharmaceuticals	Mass loads (g/d)																					Residual proportion (%)		
		Influent			Primary effluent			Secondary effluent			Tertiary effluent			Activated sludge			Return sludge			Excess sludge			Primary treatment	Secondary treatment	Tertiary treatment
		L	S	T	L	S	T	L	S	T	L	S	T	L	S	T	L	S	T	L	S	T			
A	Acetaminophen	1144	1	1145	1213	1	1214	2	0	2	-	-	-	1	0	1	0	0	0	0	0	0	106	0	-
	Acetylsalicylic acid	311	6	318	346	9	354	0	0	0	-	-	-	1	254	254	1	910	912	0	6	6	112	0	-
	Atenolol	6	1	7	6	0	6	3	0	3	-	-	-	1	0	1	0	0	0	0	0	0	84	46	-
	Caffeine	989	1	990	941	4	945	0	0	0	-	-	-	0	21	22	1	65	66	0	0	0	95	0	-
	Carbamazepine	4	0	4	4	0	4	5	0	5	-	-	-	4	0	4	2	0	2	0	0	0	108	122	-
	Cefradine	60	0	60	28	0	28	1	0	1	-	-	-	2	0	2	49	126	175	0	1	1	46	2	-
	Cimetidine	95	0	96	97	0	97	104	0	104	-	-	-	142	203	344	103	399	502	2	7	9	101	109	-
	Ciprofloxacin	10	0	10	9	0	10	5	0	5	-	-	-	4	210	214	4	295	300	0	13	13	95	48	-
	Clarithromycin	21	0	22	22	0	23	9	0	9	-	-	-	4	17	21	1	55	56	0	0	0	104	41	-
	Diclofenac	1	0	1	2	0	2	6	0	6	-	-	-	8	17	25	7	61	68	0	0	1	136	412	-
	Diphenhydramine	3	0	3	3	0	4	6	0	6	-	-	-	3	35	38	1	123	124	0	1	1	118	178	-
	Erythromycin	1	0	1	0	0	0	0	0	0	-	-	-	0	0	0	0	0	0	0	0	0	24	15	-
	Gemfibrozil	1	0	1	1	0	1	0	0	0	-	-	-	0	0	0	0	0	0	0	0	0	85	0	-
	Ibuprofen	65	2	67	37	6	43	25	0	25	-	-	-	108	761	869	90	1860	1950	2	9	11	64	37	-
	Iopromide	30	0	30	26	0	26	7	0	7	-	-	-	5	0	5	0	0	0	0	0	0	86	24	-
	Ketoprofen	5	0	5	5	0	5	0	0	0	-	-	-	1	9	10	3	48	52	0	1	1	104	1	-
	Naproxen	86	0	87	94	0	94	1	0	1	-	-	-	1	1	2	1	3	4	0	0	0	109	2	-
	Ofloxacin	18	1	18	19	1	20	9	0	9	-	-	-	8	292	300	6	393	399	0	13	13	109	48	-
	Oxolinic acid	0	0	0	0	0	0	0	0	0	-	-	-	0	2	2	0	0	0	0	0	0	114	0	-
	Propranolol	0	0	0	0	0	0	0	0	0	-	-	-	0	7	7	0	29	29	0	0	0	-	-	-
	Roxithromycin	5	1	6	11	1	12	5	0	5	-	-	-	3	30	33	0	119	120	0	1	1	214	87	-
	Sildenafil	1	0	1	0	0	0	1	0	1	-	-	-	0	11	11	0	45	45	0	0	0	54	92	-
	Sulfadimethoxine	1	0	1	0	0	0	0	0	0	-	-	-	0	59	59	0	2	2	0	0	0	0	5	-
	Sulfamethazine	0	0	0	0	0	0	0	0	0	-	-	-	0	0	0	0	0	0	0	0	0	-	-	-
	Sulfamethoxazole	2	0	2	3	0	3	1	0	1	-	-	-	1	0	1	1	0	1	0	0	0	126	58	-
	Testosterone	1	0	1	1	0	1	0	0	0	-	-	-	0	2	2	0	7	7	0	0	0	137	10	-
	Trimethoprim	1	0	1	1	0	1	1	0	1	-	-	-	0	0	0	0	0	0	0	0	0	120	117	-

L: mass loads of pharmaceuticals in the liquid phase, S: mass loads of pharmaceuticals in the solid phase, T: total mass loads of pharmaceuticals.

Table S6. Mass loads and residual proportion of pharmaceuticals in different unit processes of STP B.

STPs	Pharmaceuticals	Mass loads (g/d)																					Residual proportion (%)		
		Influent			Primary effluent			Secondary effluent			Tertiary effluent			Activated sludge			Return sludge			Excess sludge			Primary treatment	Secondary treatment	Tertiary treatment
		L	S	T	L	S	T	L	S	T	L	S	T	L	S	T	L	S	T	L	S	T			
B	Acetaminophen	3066	2	3069	3565	2	3567	2	0	2	2	0	2	2	16	18	1	6	6	0	0	0	116	0	0
	Acetylsalicylic acid	612	7	619	549	9	558	2	5	7	1	7	8	2	229	231	1	133	134	0	5	5	90	1	1
	Atenolol	21	0	21	23	0	23	21	0	21	20	0	20	17	2	19	3	0	3	0	0	0	108	98	94
	Caffeine	1196	1	1197	1120	1	1121	1	0	1	1	2	3	0	9	9	0	7	7	0	0	0	94	0	0
	Carbamazepine	15	0	15	16	0	16	14	0	14	14	0	14	14	0	14	2	0	2	0	0	0	106	94	95
	Cefradine	170	0	170	185	1	186	4	8	11	2	0	2	107	138	244	1	1	2	0	0	0	109	7	1
	Cimetidine	287	0	287	279	0	279	263	1	263	258	0	258	199	43	242	88	201	289	3	8	11	97	92	90
	Ciprofloxacin	12	1	13	10	0	10	4	0	4	4	0	4	3	47	50	1	194	196	0	8	8	78	33	33
	Clarithromycin	51	3	55	56	1	57	52	1	53	51	1	51	47	91	138	5	46	51	0	2	2	105	97	94
	Diclofenac	3	0	3	4	0	4	19	0	19	20	0	20	28	4	32	7	9	17	0	0	1	122	568	594
	Diphenhydramine	8	0	8	9	0	9	12	0	12	11	0	12	6	42	48	1	46	47	0	2	2	107	148	143
	Erythromycin	1	0	1	1	0	1	3	0	3	3	0	3	2	0	2	0	0	0	0	0	0	93	366	367
	Gemfibrozil	2	0	2	3	0	3	2	0	2	1	0	2	1	1	2	0	1	1	0	0	0	129	76	73
	Ibuprofen	159	4	162	166	6	171	142	2	144	170	6	176	248	159	407	68	143	211	3	6	8	106	89	108
	Iopromide	604	1	606	473	2	475	141	1	142	133	1	134	89	2	90	18	19	37	1	1	1	78	24	22
	Ketoprofen	34	0	34	30	0	31	16	0	16	16	0	16	16	0	16	3	0	3	0	0	0	90	48	46
	Naproxen	221	0	221	233	0	233	10	0	10	10	0	10	5	2	7	1	1	1	0	0	0	105	4	4
	Ofloxacin	18	1	18	17	0	17	9	0	9	9	0	9	7	58	65	2	141	143	0	6	6	95	49	49
	Oxolinic acid	0	0	0	0	0	0	0	0	0	0	0	0	0	0	0	0	1	2	0	0	0	-	-	-
	Propranolol	1	0	1	1	0	1	4	0	4	3	0	3	1	4	5	0	7	8	0	0	0	118	421	379
	Roxithromycin	20	0	21	23	2	25	23	0	23	21	1	22	18	33	51	2	17	19	0	1	1	122	112	106
	Sildenafil	1	0	1	1	0	1	1	0	1	1	0	1	0	7	7	0	8	8	0	0	0	98	116	108
	Sulfadimethoxine	1	0	1	3	0	3	0	0	0	0	0	0	0	0	0	0	9	9	0	0	0	404	26	12
	Sulfamethazine	0	0	0	0	0	0	0	0	0	0	0	0	0	0	0	0	0	0	0	0	0	-	-	-
	Sulfamethoxazole	20	0	20	20	0	20	12	0	12	12	0	12	11	1	12	2	4	6	0	0	0	101	62	61
	Testosterone	0	0	0	0	0	0	0	0	0	0	0	0	1	3	4	0	1	1	0	0	0	-	-	-
	Trimethoprim	6	0	6	6	0	6	8	0	8	7	0	7	4	0	4	1	0	1	0	0	0	101	131	128

L: mass loads of pharmaceuticals in the liquid phase, S: mass loads of pharmaceuticals in the solid phase, T: total mass loads of pharmaceuticals.

Table S7. Mass loads and residual proportion of pharmaceuticals in different unit processes of STP C.

STPs	Pharmaceuticals	Mass loads (g/d)																					Residual proportion (%)		
		Influent			Primary effluent			Secondary effluent			Tertiary effluent			Activated sludge			Return sludge			Excess sludge			Primary treatment	Secondary treatment	Tertiary treatment
		L	S	T	L	S	T	L	S	T	L	S	T	L	S	T	L	S	T	L	S	T			
C	Acetaminophen	1155	1	1157	1375	1	1376	2	0	2	0	0	0	1	7	8	5	0	5	0	0	0	119	0	0
	Acetylsalicylic acid	262	4	265	242	3	246	2	2	4	2	2	5	3	108	110	6	1201	1207	0	4	4	93	1	2
	Atenolol	9	0	9	9	0	9	12	0	12	10	0	10	10	2	12	8	2	10	0	0	0	98	131	118
	Caffeine	832	1	833	740	1	741	0	0	0	0	0	0	0	4	4	0	111	111	0	0	0	89	0	0
	Carbamazepine	9	0	9	9	0	9	9	0	9	9	0	9	8	1	9	15	22	37	0	0	0	105	101	101
	Cefradine	38	0	38	62	0	62	1	0	1	0	0	0	0	22	22	1	0	1	0	0	0	164	2	1
	Cimetidine	117	0	117	129	0	129	85	0	85	80	0	80	54	31	85	296	255	552	1	1	3	111	73	68
	Ciprofloxacin	2	0	2	2	0	2	1	0	1	1	0	1	2	11	13	5	92	96	0	0	0	102	61	75
	Clarithromycin	34	1	35	37	1	38	55	0	55	54	1	55	44	85	129	56	710	766	0	2	3	109	159	158
	Diclofenac	2	0	2	3	0	3	12	0	12	12	0	12	15	13	28	34	151	185	0	1	1	120	546	557
	Diphenhydramine	4	0	4	5	0	5	8	0	8	8	0	8	5	31	36	8	445	453	0	2	2	117	195	187
	Erythromycin	1	0	1	1	0	1	3	0	3	3	0	3	2	2	4	3	14	18	0	0	0	134	315	316
	Gemfibrozil	1	0	1	1	0	1	0	0	0	0	0	0	0	1	1	1	11	12	0	0	0	136	43	45
	Ibuprofen	60	2	62	17	1	18	65	0	65	59	1	60	68	161	228	257	1447	1705	1	6	8	29	105	96
	Iopromide	42	0	42	35	0	35	15	0	15	15	0	15	11	0	11	32	23	55	0	0	0	83	36	36
	Ketoprofen	15	0	15	15	0	15	4	0	4	4	0	4	3	3	6	3	2	5	0	0	0	100	25	27
	Naproxen	103	0	103	111	0	111	1	0	1	1	0	1	1	2	2	3	17	19	0	0	0	108	1	1
	Ofloxacin	6	0	6	6	0	6	4	0	4	2	0	2	2	162	165	8	273	280	0	1	1	105	62	29
	Oxolinic acid	0	0	0	0	0	0	0	0	0	0	0	0	0	2	3	0	12	12	0	0	0	-	-	-
	Propranolol	1	0	1	1	0	1	2	0	2	2	0	2	1	7	7	2	65	67	0	0	0	119	376	400
	Roxithromycin	17	0	18	19	0	19	25	1	25	23	0	24	19	40	59	29	294	323	0	1	1	111	146	136
	Sildenafil	0	0	0	0	0	0	0	0	0	0	0	0	0	4	4	0	46	46	0	0	0	-	-	-
	Sulfadimethoxine	0	0	0	0	0	0	0	0	0	0	0	0	0	1	1	1	2	3	0	0	0	-	-	-
	Sulfamethazine	0	0	0	0	0	0	0	0	0	0	0	0	0	0	0	0	0	0	0	0	0	-	-	-
	Sulfamethoxazole	6	0	6	7	0	7	3	0	3	3	0	3	2	3	6	5	20	25	0	0	0	111	52	55
	Testosterone	0	0	0	0	0	0	0	0	0	0	0	0	0	2	3	1	10	12	0	0	0	-	-	-
	Trimethoprim	2	0	2	2	0	2	2	0	2	2	0	2	1	0	1	2	0	2	0	0	0	103	134	130

L: mass loads of pharmaceuticals in the liquid phase, S: mass loads of pharmaceuticals in the solid phase, T: total mass loads of pharmaceuticals.

Table S8. Mass loads and residual proportion of pharmaceuticals in different unit processes of STP D.

STPs	Pharmaceuticals	Mass loads (g/d)																					Residual proportion (%)		
		Influent			Primary effluent			Secondary effluent			Tertiary effluent			Activated sludge			Return sludge			Excess sludge			Primary treatment	Secondary treatment	Tertiary treatment
		L	S	T	L	S	T	L	S	T	L	S	T	L	S	T	L	S	T	L	S	T			
D	Acetaminophen	1458	5	1462	1738	3	1741	1	0	1	1	0	1	2	0	2	1	0	1	0	0	0	119	0	0
	Acetylsalicylic acid	130	7	137	146	5	151	1	2	2	1	2	2	1	150	151	1	154	156	0	2	2	110	2	2
	Atenolol	9	0	9	11	0	11	6	0	6	5	0	5	5	0	5	1	0	1	0	0	0	120	68	52
	Caffeine	2242	11	2253	2159	5	2164	1	0	1	1	0	1	1	15	15	0	29	29	0	0	0	96	0	0
	Carbamazepine	5	0	5	7	0	7	7	0	7	6	0	6	6	0	6	3	8	11	0	0	0	142	140	117
	Cefradine	101	0	101	81	0	81	1	0	1	0	0	0	0	0	0	0	0	0	0	0	0	80	1	0
	Cimetidine	135	0	135	142	0	143	63	0	63	44	0	44	85	34	119	74	114	188	1	2	3	106	47	33
	Ciprofloxacin	6	0	6	6	0	7	8	0	8	4	0	5	6	19	25	5	54	58	0	1	1	102	121	71
	Clarithromycin	22	2	24	29	1	30	37	1	38	32	0	33	28	49	77	6	204	210	0	3	3	124	156	135
	Diclofenac	4	0	4	5	0	5	19	0	19	16	0	16	23	16	39	14	73	87	0	1	1	116	480	404
	Diphenhydramine	2	0	2	3	0	3	6	0	6	4	0	4	3	15	18	1	66	67	0	1	1	145	303	224
	Erythromycin	1	0	1	1	0	1	1	0	1	1	1	2	1	0	1	0	1	1	0	0	0	96	196	317
	Gemfibrozil	2	0	2	4	0	4	1	0	1	1	0	1	1	1	2	1	6	7	0	0	0	182	55	31
	Ibuprofen	590	3	593	890	3	893	22	1	23	33	1	34	50	35	85	53	179	231	1	3	3	151	4	6
	Iopromide	55	0	56	40	0	40	27	0	27	25	0	25	35	2	37	16	17	33	0	0	0	71	49	44
	Ketoprofen	8	0	8	8	0	9	5	0	5	2	0	2	5	2	7	2	10	12	0	0	0	109	66	30
	Naproxen	125	0	125	127	0	128	7	0	7	7	0	7	11	1	13	3	7	10	0	0	0	102	6	6
	Ofloxacin	9	0	9	6	0	6	4	0	4	2	1	3	4	25	29	3	45	48	0	1	1	65	44	38
	Oxolinic acid	0	0	0	0	0	0	0	0	0	0	0	0	0	0	0	0	7	7	0	0	0	-	-	-
	Propranolol	0	0	0	0	0	0	0	0	0	0	0	0	0	3	3	0	16	16	0	0	0	-	-	-
	Roxithromycin	13	1	14	15	0	15	18	7	25	14	2	16	16	17	32	3	72	74	0	1	1	109	175	111
	Sildenafil	0	0	0	0	0	0	0	0	0	0	0	0	0	0	0	0	11	11	0	0	0	-	-	-
	Sulfadimethoxine	0	0	0	0	0	0	0	0	0	0	0	0	0	0	0	0	0	0	0	0	0	-	-	-
	Sulfamethazine	0	0	0	0	0	0	0	0	0	0	0	0	0	0	0	0	0	0	0	0	0	-	-	-
	Sulfamethoxazole	2	0	2	3	0	3	2	0	2	2	0	2	2	1	3	1	8	10	0	0	0	117	98	99
	Testosterone	1	0	2	1	0	1	0	0	0	0	0	0	0	2	3	0	8	8	0	0	0	84	17	13
	Trimethoprim	1	0	1	2	0	2	3	0	3	2	0	2	1	0	1	1	0	1	0	0	0	137	186	127

L: mass loads of pharmaceuticals in the liquid phase, S: mass loads of pharmaceuticals in the solid phase, T: total mass loads of pharmaceuticals.

Table S9. Standardized removal efficiencies of pharmaceuticals in biological and tertiary treatment processes.

	Biological treatment processes				Tertiary treatment processes				
	MBR	SBR	A2O	MBBR	CD	CS	RCS	UV	PAC
Acetaminophen	-0.251	0.151	-0.17	0.27	0.0542	0.3063	-0.2344	-0.0768	-0.0493
Acetylsalicylic acid	0.354	-0.012	-0.126	-0.216	0.1426	-0.1780	0.2796	-0.1242	-0.1200
Atenolol	0.185	-0.039	-0.339	0.193	-0.1794	0.0049	-0.1036	-0.0589	0.3370
Caffeine	-0.033	-0.341	0.179	0.195	0.0680	0.1158	0.0425	-0.3525	0.1262
Carbamazepine	-0.346	0.245	0.086	0.016	0.0850	0.0129	0.2579	-0.0711	-0.2847
Cefradine	-0.339	-0.039	0.193	0.184	0.3536	-0.0729	-0.0986	-0.1321	-0.0500
Cimetidine	-0.26	-0.143	0.108	0.296	-0.1144	-0.0648	-0.0741	-0.1026	0.3559
Ciprofloxacin	0.044	0.23	0.082	-0.355	-0.0260	-0.1965	0.3355	-0.0269	-0.0861
Clarithromycin	0.239	0.187	-0.263	-0.163	-	-	-	-	-
Diclofenac	0.354	-0.203	-0.145	-0.006	-	-	-	-	-
Diphenhydramine	0.132	0.205	0.019	-0.357	-	-	-	-	-
Gemfibrozil	0.299	-0.313	-0.001	0.015	-0.0050	-0.0623	-0.2228	-0.0343	0.3245
Ibuprofen	0.105	0.071	-0.367	0.191	-0.1602	-0.0052	-0.0813	-0.0971	0.3439
Iopromide	0.137	0.237	-0.041	-0.333	0.1627	0.1080	-0.2091	0.1644	-0.2261
Ketoprofen	0.306	-0.143	0.092	-0.255	-0.0269	-0.0999	-0.1807	-0.0324	0.3400
Naproxen	0.185	-0.161	0.24	-0.264	0.1290	0.1206	-0.3082	0.1560	-0.0973
Ofloxacin	0.303	0.075	-0.092	-0.286	-0.1314	0.3272	0.0569	-0.1110	-0.1417
Roxithromycin	0.282	0.125	-0.134	-0.272	-	-	-	-	-
Sulfamethoxazole	0.187	-0.019	0.179	-0.347	0.1616	0.0748	-0.0449	0.1365	-0.3281
Trimethoprim	0.372	-0.136	-0.08	-0.156	-0.1417	-0.0544	-0.0262	-0.1251	0.3473
Average SREs	0.113	-0.001	-0.029	-0.083	0.023	0.021	-0.038	-0.056	0.049

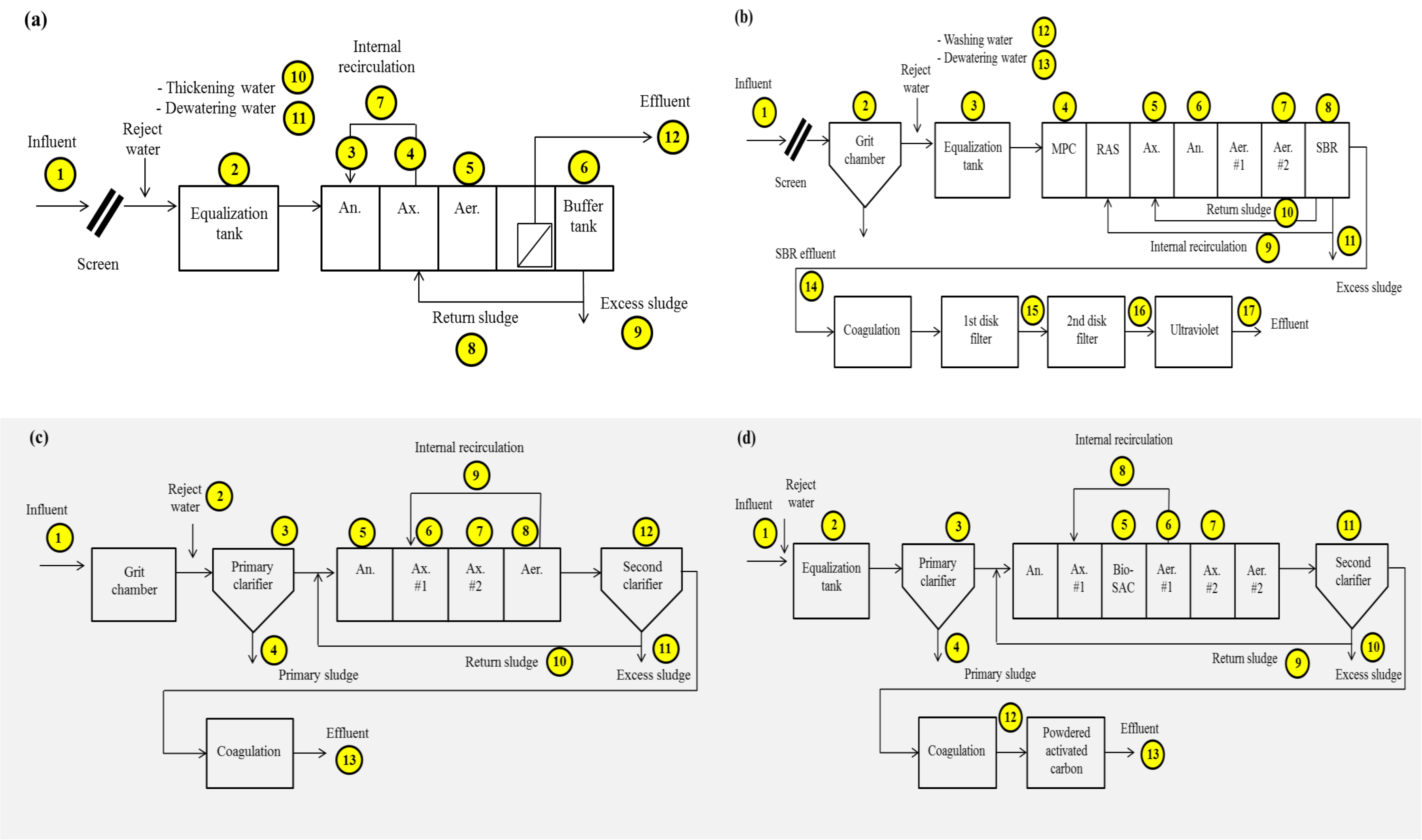

Fig. S1. Process flow diagrams and sampling sites of target STPs. (a) STP A, (b) STP B, (c) STP C, (d) STP D.; An.: anaerobic tank, Ax.: anoxic tank, Aer.: aerobic tank, MPC: modified primary clarifier, RAS: return activated sludge tank, SBR: sequencing batch reactor.

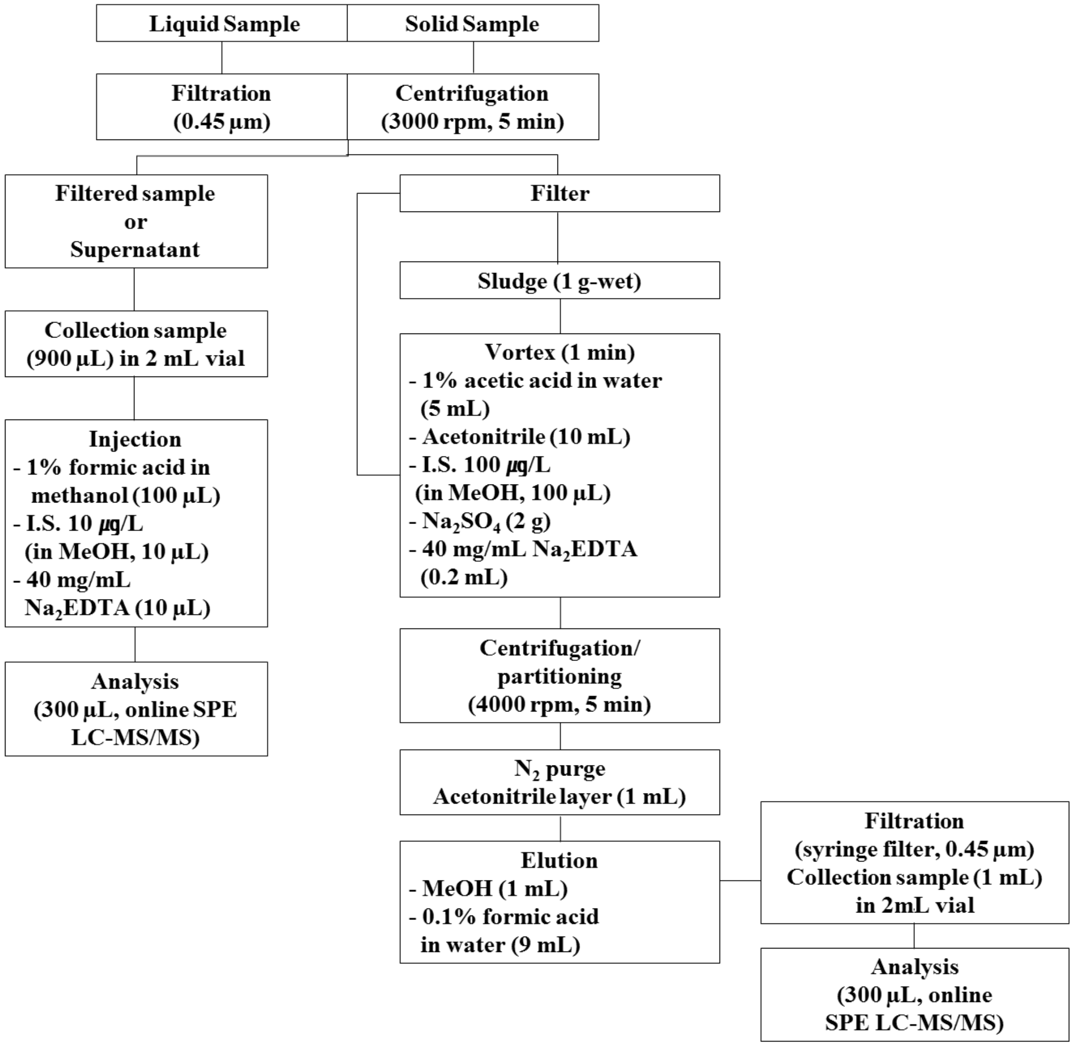

Fig. S2. Schematic diagram of the pharmaceutical analysis for liquid and solid samples.

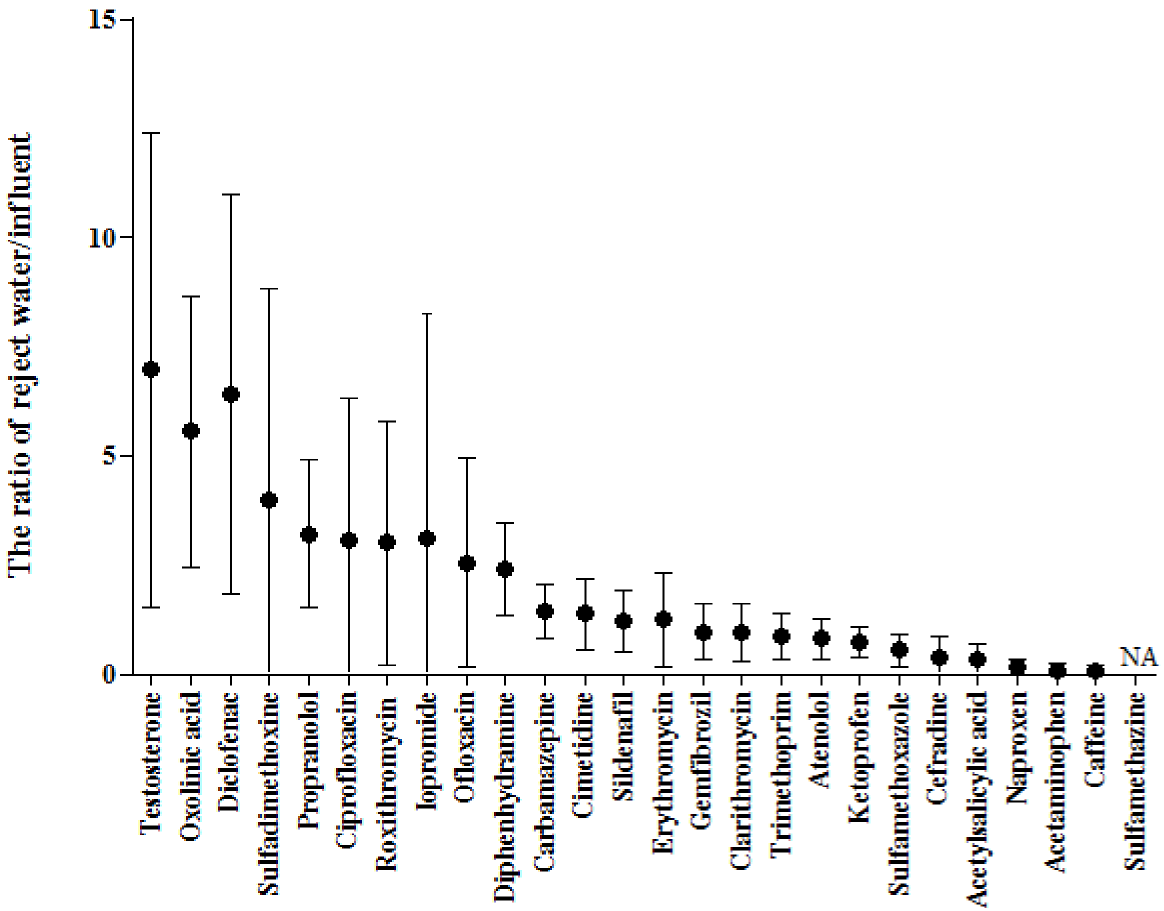

Fig. S3. Ratio of reject water to influent of pharmaceuticals in the studied STPs. Among 27 target pharmaceuticals, 26 compounds are indicated in the figure, except for ibuprofen (The ratio for ibuprofen is 24.3). NA: not available data.

[1] Park, J.; Kim, C.; Ju, B.; Lee, W.; Chung, H.; Jeong, D. Evaluation and application of pretreatment methods for pharmaceuticals and personal care products in the solid phase of sewage samples, *J. Korean Soc. Water Wastewater* **2018**, 32(6), 559-572.

[2] Anastassiades, M.; Lehotay, S.J.; Stajnbaher, D.; Schenck, F.J. Fate and easy multiresidue method employing acetonitrile extraction/partitioning and "dispersive solid-phase-extraction" for the determination of pesticide residues in produce. *J. AOAC Int.* **2003**, 86(2), 412–431.

[3] Association of Analytical Communities (AOAC) International AOAC Official Method 2007.01 pesticide residues in foods by acetonitrile extraction and partitioning with magnesium sulfate. *Off. Methods Anal. AOAC Int.* **2011**, 90, 17–26.

[4] European Committee for Standardization (CEN) Foods of plant origin – determination of pesticide residues using GC-MS and/or LC-MS/MS following acetonitrile extraction/partitioning and clean-up by dispersive SPE – QuEChERS-method. *EN 15662*, **2011**, 24, 1–83.

[5] Guo, C.; Wang, M.; Xiao, H.; Huai, B.; Wang, F.; Pan, G.; Liao, X.; Liu, Y. Development of a modified QuEChERS method for the determination of veterinary antibiotics in swine manure by liquid chromatography tandem mass spectrometry. *J. Chromatography B* **2016**, 1027, 110–118.

[6] Ponce-Robles, L.; Rivas, G.; Esteban, B.; Oller, I.; Malato, S.; Aguera, A. Determination of pesticides in sewage sludge from an agro-food industry using QuEChERS extraction followed by analysis with liquid chromatography-tandem mass spectrometry. *Anal. Bioanal. Chem.* **2017**, 409, 6181–6193.

[7] Rossini, D.; Ciofi, L.; Ancillotti, C.; Checchini, L.; Bruzzoniti, M.C.; Rivoira, L.; Fibbi, D.; Orlandini, S.; Del Bubba, M. Innovative combination of QuEChERS extraction with on-line solid-phase extract purification and pre-concentration, followed by liquid chromatography-tandem mass spectrometry for the determination of non-steroidal anti-inflammatory drugs and their metabolites in sewage sludge. *Anal. Chim. Acta*. **2016**, 935, 269–281.

[8] Fan, Y.; Shen, G.; Li, P.; Xi, X.; Wu, H.; Tian, H.; Lu, Y.; Yin, Z. A simple and automated online SPE-LC-MS/MS method for simultaneous determination of olanzapine, fluoxetine and norfluoxetine in human plasma and its application in therapeutic drug monitoring. *RSC Adv.* **2015**, 5, 34342–34352.

[9] Kim, C.; Ryu, H.; Chung E.G.; Kim, Y. Determination of 18 veterinary antibiotics in environmental water using high-performance liquid chromatography-q-orbitrap combined with on-line solid-phase extraction. *J. Chromatogr. B* **2018**, 1084, 158–165.

[10] Hong, Y.; Lee, I.; Lee, W.; Kim, H. Mass-balance-model-based evaluation of sewage treatment plant contribution to residual pharmaceuticals in environmental waters. *Chemosphere* **2019**, 225, 378–387.
